# Mitochondrial Ca^2+^ homeostasis in ovarian function and oocyte competence: from molecular transporters to early embryonic development

**DOI:** 10.3389/fcell.2026.1963467

**Published:** 2026-09-11

**Authors:** Xiaoqian Fu, Qianyi Huang, Huimei Wu, Lidan Liu

**Affiliations:** The First Affiliated Hospital of Guangxi Medical University, Nanning, Guangxi, China

**Keywords:** embryo development, ER-mitochondria contact, fertilization, MCU, MICU1, mitochondrial Ca^2+^, NCLX, oocyte competence

## Abstract

Mitochondrial Ca^2+^ homeostasis is a critical interface connecting ovarian cell signaling, energy metabolism, redox balance, and reproductive competence. Transient Ca^2+^ uptake into the mitochondrial matrix activates Ca^2+^-sensitive dehydrogenases, enhances reducing-equivalent generation, and supports oxidative phosphorylation. By contrast, sustained Ca^2+^ accumulation promotes reactive oxygen species production, membrane-potential collapse, mitochondrial permeability transition, and cell death. The identification of the mitochondrial calcium uniporter together with its regulators MICU1, MICU2, and EMRE, has established a molecular framework for mitochondrial Ca^2+^ influx. NCLX and its interacting protein TMEM65 contribute to Ca^2+^ efflux and determine recovery after individual Ca^2+^ transients. In ovarian cells and oocytes, endoplasmic reticulum (endoplasmic reticulum)-mitochondria contact sites, including the IP_3_R1-GRP75-VDAC1 axis, couple cytosolic Ca^2+^ signals to mitochondrial metabolism. Evidence from mouse, porcine, avian, zebrafish, *Xenopus*, and sea-urchin models implicates mitochondrial Ca^2+^ in follicular-cell survival, oocyte meiotic maturation, fertilization-associated Ca^2+^ oscillations, the oocyte-to-embryo transition, and early embryonic development. Obesity, aging, cryopreservation, heavy metals, environmental chemicals, and oxidative stress can disturb this system. However, mitochondrial Ca^2+^ dysregulation is not always readily separable from broader mitochondrial or ER dysfunction. Major limitations of the current literature include reliance on non-selective pharmacological agents, incomplete calibration of organelle-targeted indicators, insufficient temporal resolution, interspecies differences, and limited direct evidence from human oocytes. Future studies should integrate cell-type-specific genetic perturbation, quantitative multi-organelle Ca^2+^ imaging, mitochondrial bioenergetics, and long-term developmental assessment. Mitochondrial Ca^2+^ is a promising mechanistic node and candidate biomarker, but it is not yet a validated clinical target in reproductive medicine.

## 1 Introduction

Ovarian function depends on coordinated communication among the oocyte, follicular somatic cells, and the endocrine environment. Within the follicle, mitochondria support ATP production, redox and apoptotic regulation, steroidogenesis, and Ca^2+^-metabolic coupling across oocyte growth, meiotic maturation, fertilization, and early cleavage. Mitochondrial abnormalities present at the end of follicular growth may therefore compromise maturation, fertilization, or early embryonic development (Van Blerkom, 2011).

The reproductive importance of mitochondria extends beyond ATP production: embryos inherit their mitochondrial population almost entirely from the oocyte, and mtDNA replication is limited during preimplantation development (Van Blerkom, 2011). Mitochondrial mass, morphology, distribution, membrane potential, respiratory capacity, mtDNA integrity, and redox buffering represent distinct dimensions of mitochondrial status.

Mitochondrial ATP production, membrane potential, spatial organization, and mtDNA content have each been associated with oocyte or embryo developmental competence (Van Blerkom, 2011). However, clinically applicable, non-invasive methods for assessing these properties in living human oocytes remain underdeveloped. Current assessment still relies predominantly on morphological observation, polar-body evaluation, and, in selected settings, spindle imaging (Boylan et al., 2024).

Ca^2+^ adds an important regulatory dimension because it can function as a second messenger, a metabolic activator, and a trigger of cell death. Physiological, transient increases in mitochondrial matrix Ca^2+^ directly stimulate isocitrate and alpha-ketoglutarate dehydrogenases and indirectly activate pyruvate dehydrogenase through Ca^2+^-sensitive pyruvate dehydrogenase phosphatase, increasing reducing-equivalent supply to the respiratory chain and supporting oxidative phosphorylation (Szabadkai et al., 2001; Kamer and Mootha, 2015). This coupling allows mitochondrial ATP production to respond rapidly to cellular demand.

By contrast, excessive or sustained matrix Ca^2+^ loading can disrupt oxidative metabolism and promote cell death (Kamer and Mootha, 2015; Paillard et al., 2018). Mitochondrial Ca^2+^ is therefore neither intrinsically beneficial nor intrinsically harmful; its effect depends on signal amplitude, duration, spatial origin, recovery kinetics, and the cell’s energetic and antioxidant reserve.

The ovarian follicle shows a metabolic division of labour: gap junctions support metabolic cooperation between cumulus cells and the oocyte; cumulus cells metabolize glucose and supply glycolytic products such as pyruvate and lactate, which the oocyte uses for mitochondrial ATP production (Del Bianco et al., 2024).

Because cumulus-oocyte coupling integrates substrate transfer, ion-channel activity, mitochondrial metabolism, and meiotic progression, disrupted Ca^2+^ handling in either compartment may reduce oocyte developmental competence. Direct, compartment-specific measurements of mitochondrial Ca^2+^ in intact cumulus-oocyte complexes are therefore needed.

Mitochondrial Ca^2+^ homeostasis integrates uptake, buffering, metabolic utilization, and efflux. Single-endpoint measurements cannot distinguish a physiological transient from gradual matrix loading; fluorescence changes may also reflect probe loading, pH, or membrane potential (Deak et al., 2021).

Resting matrix Ca^2+^, peak amplitude, area under the curve (integrated exposure), recovery half-time, and spatial heterogeneity should therefore be distinguished experimentally. During fertilization, these measures are needed to test whether successive cytosolic Ca^2+^ transients remain discrete or become integrated when mitochondrial Ca^2+^ efflux is insufficient.

Early reproductive studies anticipated this dynamic model. In sea-urchin eggs, fertilization transiently increased mitochondrial Ca^2+^ uptake and produced measurable mitochondrial Ca^2+^ enrichment, consistent with mitochondria acting as a temporary Ca^2+^ sink during egg activation (Girard et al., 1991).

In *Xenopus* oocytes, a subset of IP_3_R-mediated local Ca^2+^-release events, or Ca^2+^ puffs, was closely associated with mitochondria. Release sites near mitochondria exhibited lower puff activity and were less likely to initiate global Ca^2+^ waves, indicating that mitochondria can regulate local ER excitability and the spatial organization of Ca^2+^ signals (Marchant et al., 2002).

Oxidizable substrates that energized mitochondria increased the amplitude and propagation velocity of IP_3_-dependent Ca^2+^ waves while prolonging the interwave interval. These effects were blocked by ruthenium red and respiratory-chain inhibitors and were associated with an increase in mitochondrial membrane potential (Jouaville et al., 1995). Taken together, the findings show that energized mitochondria regulate IP_3_-dependent Ca^2+^ wave dynamics in *Xenopus* oocytes.

**FIGURE 1:**
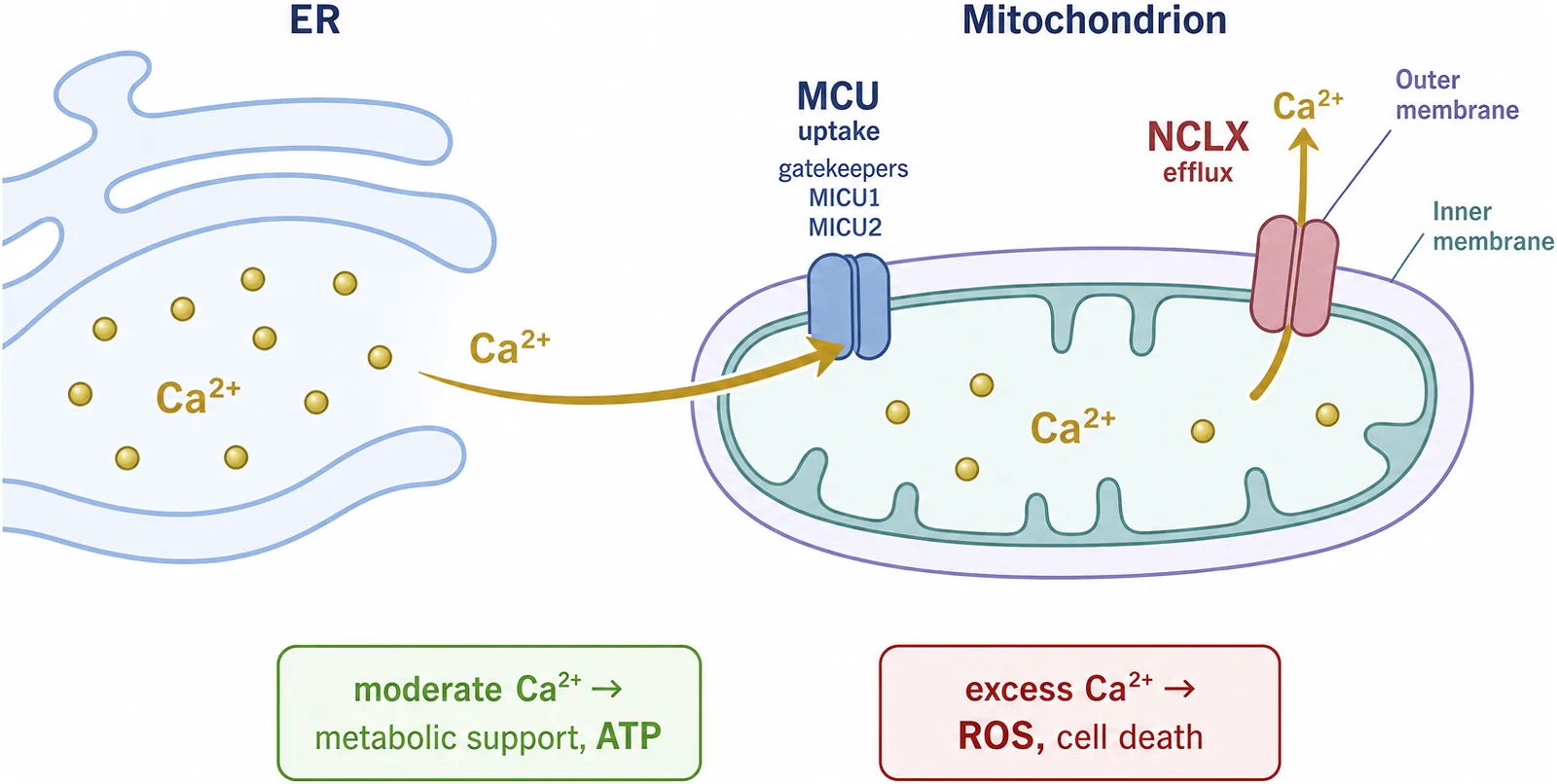
Conceptual mitochondrial Ca^2+^ transport pathway. ER-derived Ca^2+^ enters the matrix through the MCU complex, regulated by MICU1/2, and NCLX mediates efflux. Physiological uptake supports oxidative metabolism and ATP production, whereas excessive or sustained loading promotes ROS generation and cell injury. Outer-membrane transfer proteins and contact-site architecture are omitted for clarity; components are not drawn to scale.

**FIGURE 2:**
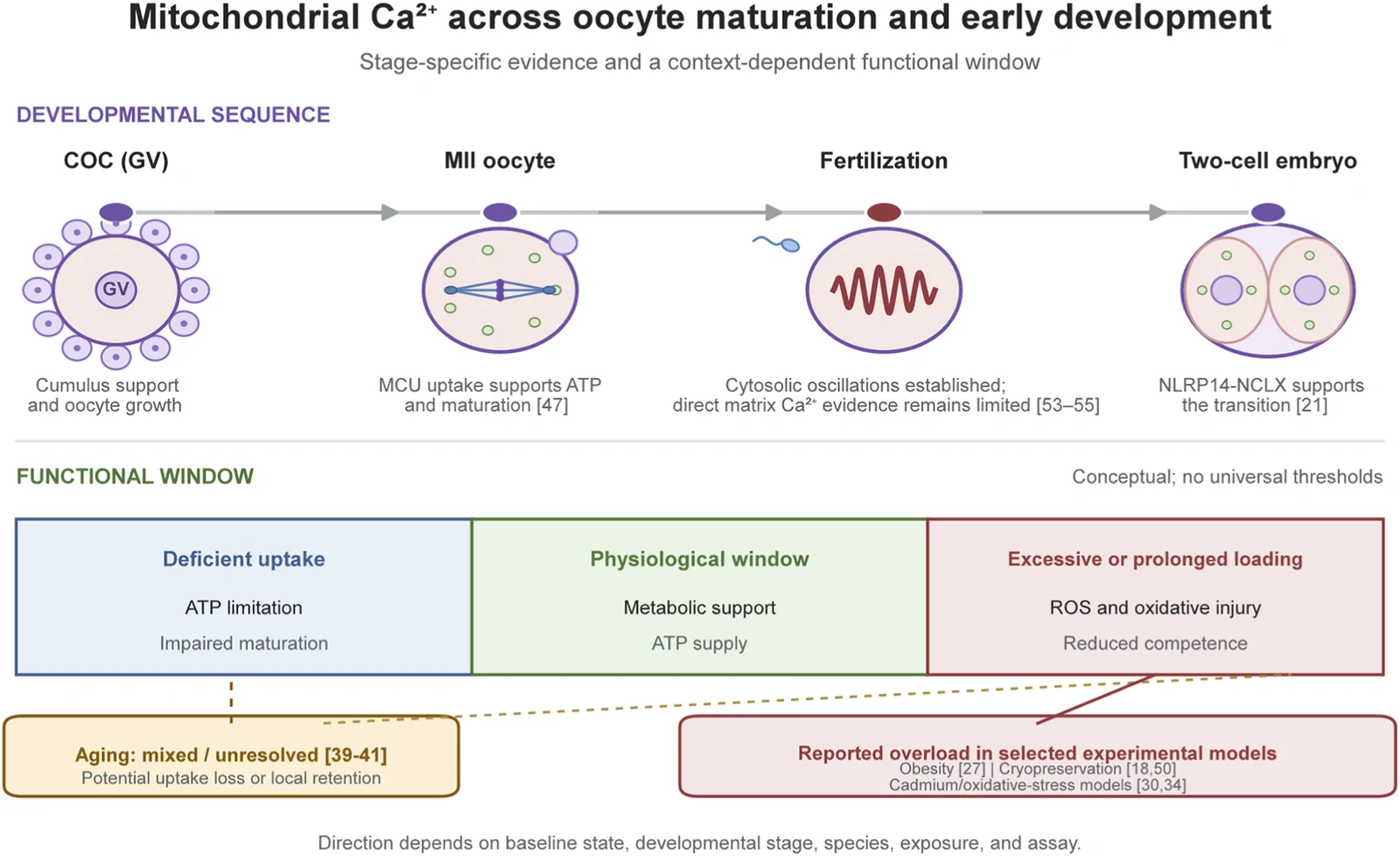
Conceptual overview of mitochondrial Ca^2+^ during oocyte maturation, fertilization, and early embryonic development. Physiological uptake supports ATP production, whereas deficient uptake or excessive loading can impair developmental competence. Selected pathological and environmental models illustrate context-dependent effects; the scheme does not define universal Ca^2+^ thresholds. Abbreviations: COC, cumulus-oocyte complex; MII, metaphase II.

**FIGURE 3:**
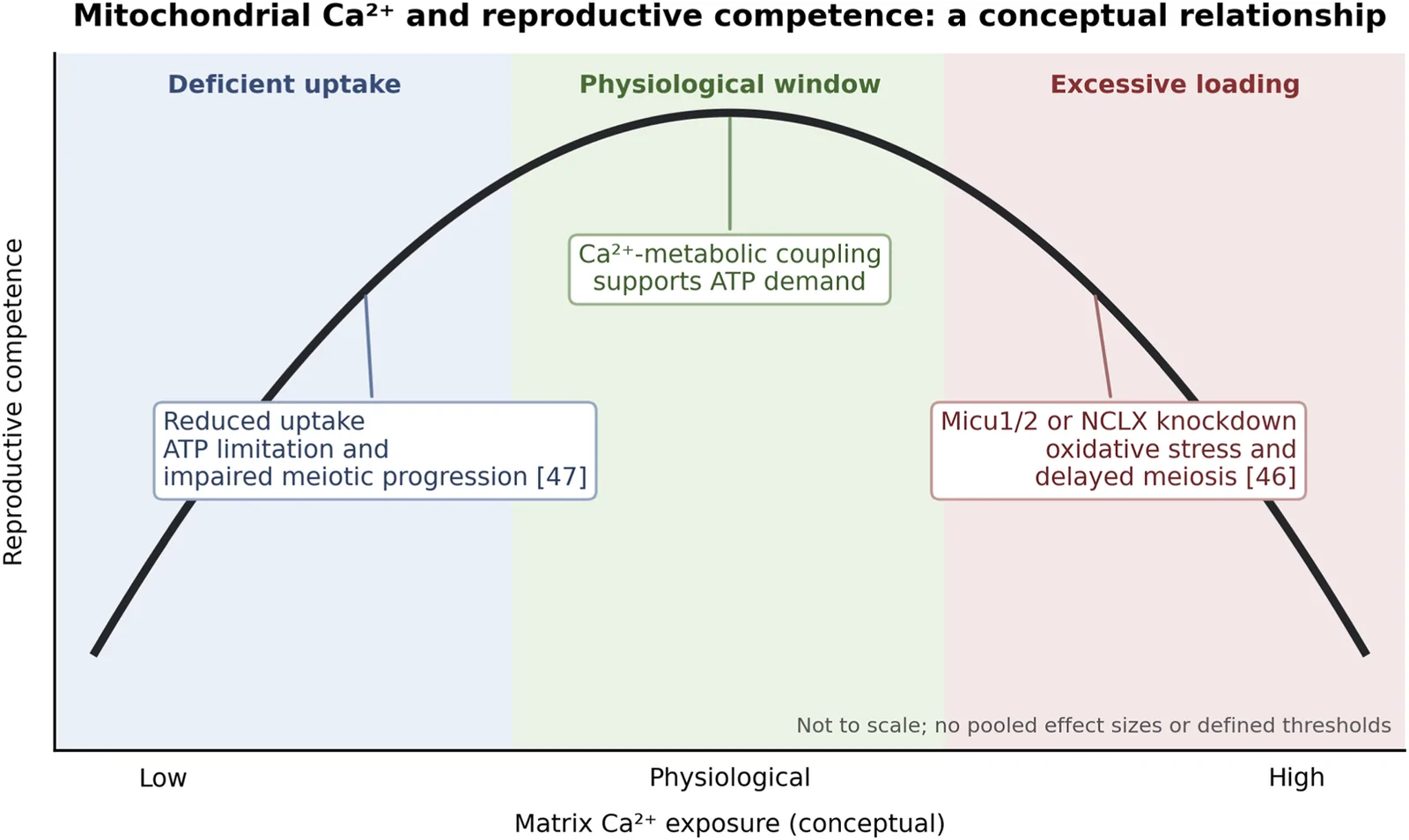
Conceptual inverted-U relationship between matrix Ca^2+^ and reproductive competence. Insufficient uptake can limit ATP production and meiotic progression, whereas excessive or prolonged loading can promote oxidative injury and developmental failure. The curve and zone boundaries are qualitative, not fitted data or defined clinical thresholds.

**FIGURE 4:**
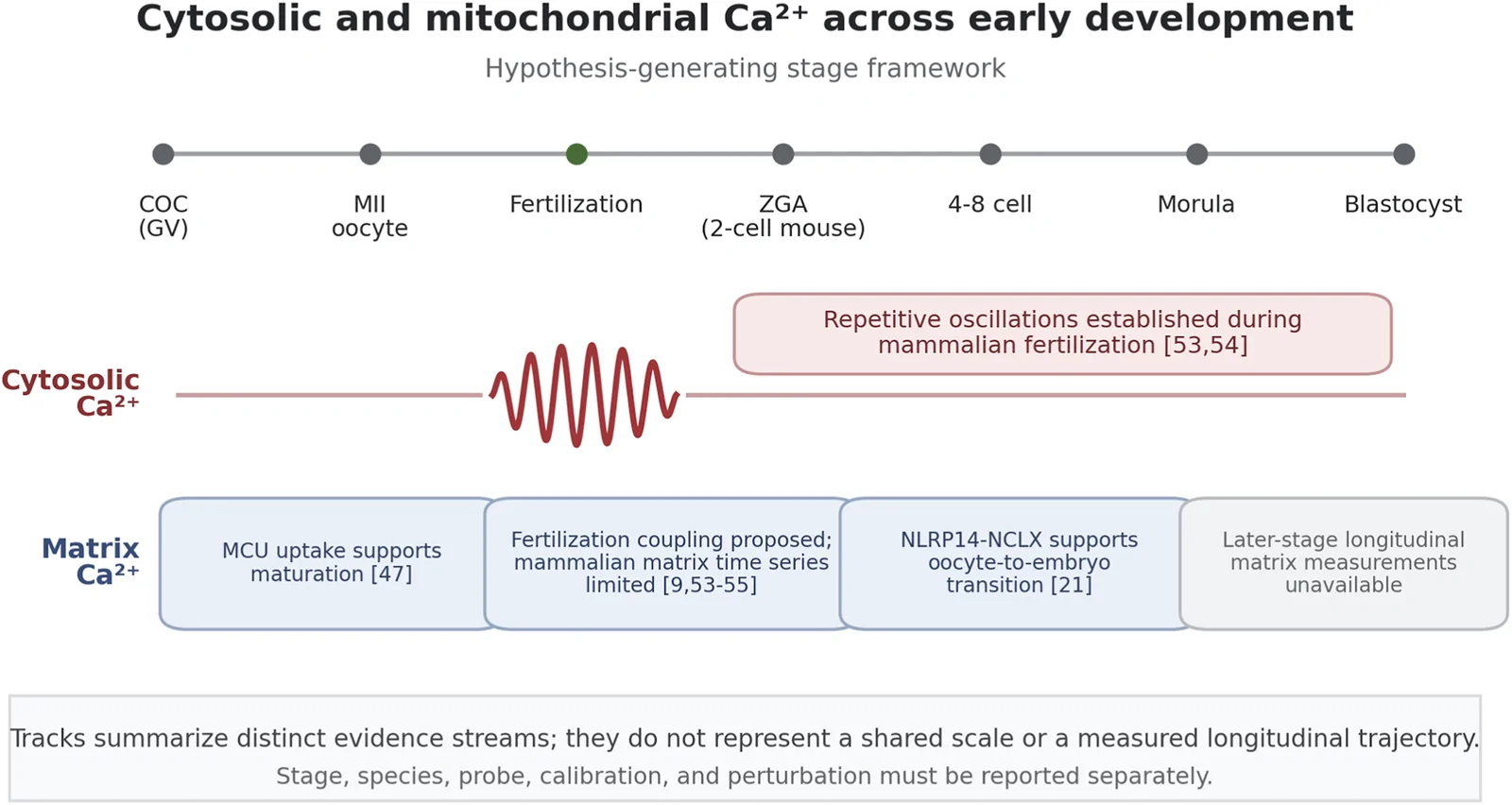
Hypothesis-generating framework for cytosolic and mitochondrial Ca^2+^ across oocyte maturation, fertilization, and preimplantation development. Fertilization-associated cytosolic oscillations are established, but comparable longitudinal matrix Ca^2+^ measurements across all stages are unavailable. Lines and relative values are illustrative rather than a dataset measured with one calibrated assay. Abbreviations: COC, cumulus-oocyte complex; MII, metaphase II; ZGA, zygotic genome activation.

Together, these studies support a dose-, time-, and context-dependent model of mitochondrial Ca^2+^ signaling across species and experimental systems. Figure 1 summarizes the core transport pathway, and Figure 2 places the proposed balance across oocyte maturation, fertilization, and early development. Figure 3 depicts the proposed non-linear relationship with reproductive competence. Figure 4 is a hypothesis-generating stage framework rather than a longitudinally measured Ca^2+^ trajectory.

## 2 Search strategy and selection criteria

This mechanism-focused narrative review was not conducted as a systematic review. Literature was identified through targeted database searching and reference-list screening to integrate molecular, cellular, and reproductive evidence rather than to estimate pooled effects.

PubMed, Scopus, and the Web of Science Core Collection were consulted using combinations of terms related to mitochondrial Ca^2+^ handling (including MCU, MICU1, MICU2, MCUb, EMRE, NCLX, TMEM65, IP_3_R1, VDAC1, GRP75, and MAMs) and reproductive contexts (including ovarian follicles, follicular cells, oocytes, fertilization, and preimplantation embryos). Searches were supplemented by backward citation screening of relevant reviews and primary studies.

Primary studies were prioritized when they examined an ovarian cell, gamete, embryo, or related reproductive context and reported at least one of the following: direct mitochondrial or cytosolic Ca^2+^ measurement; genetic, pharmacological, or electrophysiological manipulation of a mitochondrial Ca^2+^ transporter; or a reproductive outcome explicitly linked to such a measurement or manipulation. Studies reporting a reproductive outcome alone without relevant Ca^2+^ or transporter evidence were not treated as mechanistic evidence. Reviews were used for background and citation tracing rather than as independent primary evidence.

Evidence was organized by species and evaluated along two separate dimensions: experimental directness and relevance to human reproduction. Direct evidence combined transporter perturbation with mitochondrial Ca^2+^ measurement and a defined molecular, bioenergetic, or reproductive outcome. Indirect evidence relied on expression, redox, bioenergetic, or reproductive associations without transporter-specific flux measurement. Cross-species limitations are discussed in Sections 4, 6, and 10.

Peer-reviewed full articles and short reports were considered when the text was available in English or in a translation sufficient to assess the methods. Purely clinical reports without mechanistic data and papers in which mitochondrial Ca^2+^ appeared only as an unsupported discussion hypothesis were not used as mechanistic evidence. Grey-literature databases, trial registries, and dissertation repositories were not routinely searched.

The review therefore distinguishes molecular evidence obtained in non-reproductive systems from direct evidence in follicular cells, oocytes, and embryos. The central question is not simply whether mitochondrial Ca^2+^ changes during reproduction, but whether a defined transporter-dependent flux is necessary or sufficient for a specific reproductive outcome.

## 3 Molecular architecture of mitochondrial Ca^2+^ transport

### 3.1 The MCU complex

The mitochondrial calcium uniporter (MCU) is a highly Ca^2+^-selective channel complex in the inner mitochondrial membrane (Figure 1). Its activity is driven by the electrochemical potential across that membrane rather than by ATP-dependent pumping (Kamer and Mootha, 2015; Baughman et al., 2011). Integrative genomics, phylogenetic profiling, co-expression analysis, and mitochondrial proteomics identified CCDC109A, subsequently named MCU, as an essential component of the uniporter (Baughman et al., 2011).

MCU forms oligomers in the inner mitochondrial membrane, physically interacts with MICU1, and resides within a larger protein complex. MCU depletion in cultured cells and mouse liver markedly reduced mitochondrial Ca^2+^ uptake without causing an immediate loss of respiration or membrane potentia (Baughman et al., 2011).

The MCU pore contains a conserved DIME motif that contributes to ion selectivity. Mutation of residues within or adjacent to this region alters transport activity and sensitivity to ruthenium-based inhibitors (Baughman et al., 2011; Tsai and Tsai, 2018). EMRE is required for functional MCU activity in metazoans and couples the pore to its regulatory machinery. MCUb is a less conductive or inhibitory paralogue that can reduce overall uniporter activity (Kamer and Mootha, 2015; Tsai and Tsai, 2018).

The MCU:MCUb ratio, EMRE abundance, and the stoichiometry of MICU regulatory proteins may contribute to uptake capacity in ways that are not fully captured by total MCU expression. These properties have not been systematically mapped across follicular growth, meiotic maturation, fertilization, and early embryonic development.

Electrophysiological reconstruction in *Xenopus* oocytes provided direct evidence for MCU/EMRE channel activity. Plasma-membrane-targeted human MCU and EMRE produced inwardly rectifying Ca^2+^ currents that were inhibited by Ru360. Mutations that disrupted MCU-EMRE interactions or a Ca^2+^-binding site within the pore abolished the recorded currents (Tsai and Tsai, 2018).

This heterologous system permits quantitative analysis of channel conductance, ion selectivity, inhibitor sensitivity, and structure-function relationships independently of secondary mitochondrial metabolic changes. However, a plasma membrane does not reproduce the lipid environment, electrochemical potential, protein composition, or local Ca^2+^ microdomains of the mitochondrial inner membrane (Baughman et al., 2011).

The electrochemical basis of uptake is especially relevant in oocytes. Loss of mitochondrial membrane potential, whether caused by respiratory dysfunction or other insults, can reduce Ca^2+^ uptake even when cytosolic Ca^2+^ is elevated and can coincide with reduced ATP production. Matrix Ca^2+^ stimulates oxidative metabolism, whereas respiratory activity maintains the membrane potential that drives Ca^2+^ entry (Kamer and Mootha, 2015; Baughman et al., 2011).

Moderate matrix Ca^2+^ uptake supports metabolism, whereas excessive or prolonged uptake promotes oxidative injury and cell death (Kamer and Mootha, 2015). In mouse oocytes, MCU-dependent uptake has been associated with meiotic progression, whereas mitochondrial Ca^2+^ overload delays meiotic resumption (Zhang et al., 2020; Zhang et al., 2021). These findings support a time- and dose-dependent interpretation of MCU activity rather than its classification as uniformly protective or harmful.

Table 1 summarizes the core molecular evidence for the mitochondrial Ca^2+^ transport machinery and its principal functional interpretation.

**TABLE 1:** Core molecular evidence.

Biological question	Model basis and transfer limits	Representative evidence	Interpretation
Identity of MCU	Cultured mammalian cells and mouse liver; the core mechanism is broadly conserved	MCU identification and functional depletion (Baughman et al., 2011)	MCU is an essential core component of mitochondrial Ca^2+^ uptake
MCU conductance	Heterologous Xenopus-oocyte plasma-membrane system; confirmation in the native inner membrane remains necessary	MCU/EMRE electrophysiological reconstruction (Tsai and Tsai, 2018)	MCU and EMRE form a Ca^2+^-conducting complex
MICU gating	Mammalian cell and structural studies; reproductive-stage stoichiometry remains unresolved	MICU1/2 functional and structural studies (Paillard et al., 2018; Kamer et al., 2019; Matesanz-Isabel et al., 2016)	Uniporter gating is Ca^2+^-dependent and non-linear
Ca^2+^ efflux	Cardiomyocyte, muscle, structural, and mouse-oocyte evidence; direct reproductive flux data remain limited	NLRP14-NCLX, TMEM65-NCLX, and NCLX structural studies (Fan et al., 2025; Garbincius et al., 2025; Meng et al., 2023)	Efflux helps shape recovery kinetics and cumulative matrix loading
Contact-site transfer	Porcine-oocyte and non-reproductive mammalian studies; contact abundance is not equivalent to Ca^2+^ flux	IP3R1-GRP75-VDAC1 studies (Zhang et al., 2024; Yuan et al., 2022)	ER-mitochondria coupling links Ca^2+^ release to metabolism and oxidative injury
Ca^2+^ imaging	Multiple cell and animal models; oocyte-specific targeting and calibration are required	CEPIA, red CEPIA, and split indicators (Suzuki et al., 2014; Kanemaru et al., 2020; Olszakier et al., 2025)	Spatially resolved measurement is possible, but calibration is essential

### 3.2 MICU1 and MICU2 as Ca^2+^-dependent gatekeepers

MICU1 and MICU2 are EF-hand-containing Ca^2+^-sensing proteins on the intermembrane-space side of the MCU complex. At low cytosolic Ca^2+^, the MICU1-MICU2 regulatory complex suppresses MCU opening; Ca^2+^ binding to the EF-hands at higher concentrations relieves gatekeeping and promotes conductance. In HeLa and permeabilized-cell systems, MICU2 behaves predominantly as an inhibitor at low Ca^2+^, with Ca^2+^ binding lifting this brake on MCU conductance (Kamer et al., 2019; Matesanz-Isabel et al., 2016).

MICU1 and MICU2 are non-redundant, and loss of MICU1 can reduce MICU2 association with the complex (Matesanz-Isabel et al., 2016). MICU1 also interacts with the D-ring formed by the DIME motifs of MCU, contributing to gatekeeping and cooperative activation (Paillard et al., 2018). Whether MICU-dependent gatekeeping determines how local cytosolic Ca^2+^ signals are decoded in oocytes remains to be established.

### 3.3 NCLX and TMEM65-mediated Ca^2+^ efflux

NCLX, encoded by SLC8B1, has been widely regarded as a major mitochondrial Ca^2+^ efflux pathway in many mammalian cells (Kamer and Mootha, 2015; Fan et al., 2025; Garbincius et al., 2025). By promoting recovery after Ca^2+^ uptake, NCLX-dependent transport can limit cumulative matrix loading and help preserve mitochondrial function. Rather than acting only as a protective drain, this efflux may shape recovery kinetics after individual uptake events; its potential consequences for decoding fertilization-associated oscillations are discussed in Section 7.1.

The NLRP14-NCLX pathway provides direct reproductive evidence. Maternal Nlrp14 deficiency reduced NCLX abundance, altered mitochondrial distribution and morphology, and produced 2-cell embryonic arrest (Meng et al., 2023). NLRP14 interacts with an intrinsically disordered region of NCLX and regulates its K27-linked ubiquitination and stability. Exogenous Nclx mRNA reduced embryonic mortality after parthenogenetic activation but did not restore development to the 2-cell stage (Meng et al., 2023).

TMEM65 has been identified as an NCLX-binding protein that enhances Na^+^-dependent mitochondrial Ca^2+^ efflux (Garbincius et al., 2025). Pharmacological NCLX inhibition or genetic loss of NCLX abolished the TMEM65-dependent increase in efflux (Garbincius et al., 2025). This evidence was obtained mainly in cardiomyocyte and muscle models; whether TMEM65 has the same regulatory function in follicular cells, oocytes, or embryos is unknown.

The ionic mechanism of NCLX is currently unsettled. The TMEM65 study supports Na^+^-dependent exchange (Garbincius et al., 2025); a subsequent cryo-EM study proposed H^+^ as the counter-ion (Fan et al., 2025). These findings have not yet been reconciled and may reflect intact-cell versus reconstituted systems, ionic conditions, or the contribution of accessory proteins.

### 3.4 Outer-membrane transfer and whole-cell Ca^2+^ balance

Before reaching MCU, Ca^2+^ must cross the outer mitochondrial membrane. VDAC1 provides a major permeation route and is functionally coupled to ER IP_3_ receptors via GRP75 (Zhang et al., 2024). After crossing the outer membrane, Ca^2+^ enters the matrix through MCU, where phosphate and other matrix ligands contribute to buffering; matrix Ca^2+^ also regulates Ca^2+^-sensitive dehydrogenases (Kamer and Mootha, 2015; Zhang et al., 2024).

Plasma-membrane and ER transporters also shape mitochondrial exposure. PMCA extrudes Ca^2+^ from the cell, SERCA refills ER stores, and plasma-membrane channels mediate influx. Oocyte-specific PMCA1 deletion increased Ca^2+^ exposure after fertilization. Offspring derived from PMCA1-deficient eggs showed altered growth and, in males, altered body composition, indicating that abnormal Ca^2+^ handling at fertilization may have long-term developmental consequences (Savy et al., 2022).

Pharmacological experiments in mouse oocytes demonstrated distinct effects of Ca^2+^ transport pathways. TRPM7 inhibition delayed germinal-vesicle breakdown, whereas pharmacological NCLX inhibition reduced germinal-vesicle breakdown and post-GVBD survival and disrupted perinuclear mitochondrial Ca^2+^ enrichment and spindle formation. By contrast, Ru360 and erastin did not significantly inhibit maturation under the tested conditions (Wang et al., 2021a). Because these conclusions rely on pharmacological inhibitors, genetic confirmation is required.

## 4 ER-mitochondria contact sites as ovarian Ca^2+^ signaling platforms

The endoplasmic reticulum (ER) is the principal intracellular Ca^2+^ store in mammalian oocytes. Its spatial organization changes markedly during oocyte growth and meiotic maturation. ER membranes redistribute around the germinal vesicle, spindle, and cortex, eventually forming structures that support fertilization-associated Ca^2+^ release (Kang et al., 2023). IP_3_R1 is the predominant IP_3_ receptor isoform in mammalian eggs and provides a major route through which sperm-derived PLCζ initiates repetitive cytosolic Ca^2+^ oscillations. Receptor abundance, clustering, localization, and sensitivity collectively influence the capacity of an egg to generate an appropriate activation signal (Kang et al., 2023; Swann, 2023; Swann, 2025).

Mitochondria positioned close to IP_3_R1-mediated release sites are exposed to Ca^2+^ microdomains that may greatly exceed concentrations in the bulk cytosol, thereby facilitating mitochondrial uptake. The resulting matrix response depends on three linked processes: Ca^2+^ supply, shaped by ER content, IP_3_R1 activity, and contact-site geometry (Zhang et al., 2024; Kang et al., 2023); mitochondrial entry, governed by VDAC1, MCU-complex gating, and membrane potential (Kamer and Mootha, 2015; Baughman et al., 2011; Tsai and Tsai, 2018); and matrix handling, including buffering and NCLX-dependent efflux (Kamer and Mootha, 2015; Fan et al., 2025; Garbincius et al., 2025).

The IP_3_R1-GRP75-VDAC1 complex is widely proposed to facilitate ER-to-mitochondria Ca^2+^ transfer at contact sites. IP_3_R1 releases Ca^2+^ from the ER, GRP75 acts as a scaffold linking IP_3_R1-associated structures to outer-membrane VDAC1, and VDAC1 permits Ca^2+^ passage across the outer mitochondrial membrane. Ca^2+^ must subsequently cross the inner mitochondrial membrane through the MCU complex before entering the matrix. Disruption at any point in this sequence can change the matrix response; consequently, increased structural contact alone should not be interpreted as evidence of increased MCU activity (Zhang et al., 2024; Yuan et al., 2022).

ER-mitochondria contact sites are commonly studied through membrane domains referred to as mitochondria-associated membranes (MAMs). In addition to Ca^2+^ transfer, these sites participate in lipid exchange, mitochondrial dynamics, metabolic signaling, autophagy, and apoptosis (Kang et al., 2023; Yuan et al., 2022). An increased number of contacts is therefore not inherently beneficial or harmful. Functional output depends on contact geometry, molecular composition, duration, and local Ca^2+^ flux. Distant contacts may fail to generate an adequate Ca^2+^ microdomain, whereas abnormally tight or persistent coupling may promote mitochondrial Ca^2+^ overload in pathological settings (Zhang et al., 2024; Zhao et al., 2017).

### 4.1 Contact-site remodeling in metabolic stress

Maternal obesity provides an important model of pathological ER-mitochondria remodeling. Oocytes from mice with high-fat-diet-induced obesity exhibited enriched MAMs, elevated mitochondrial Ca^2+^, increased apoptosis, and impaired cytoplasmic maturation. IP_3_R1 downregulation reduced mitochondrial Ca^2+^ and apoptosis and improved cytoplasmic maturation without decreasing total MAM abundance. By contrast, PACS-2 downregulation reduced MAM abundance, mitochondrial Ca^2+^, and apoptosis and improved maturation (Zhao et al., 2017).

These interventions provide functional evidence that abnormal contact-site signaling contributes to obesity-associated oocyte dysfunction, although they do not establish contact enrichment as the sole causal lesion. Because obesity also alters cellular metabolism, redox balance, and mitochondrial function (Zhao et al., 2017), these changes could modify mitochondrial Ca^2+^ uptake independently of, or in combination with, structural changes in MAMs.

The functional significance of contact enrichment should therefore be examined by combining electron microscopy or proximity-based assays with simultaneous measurements of ER, cytosolic, and mitochondrial Ca^2+^. Lipid-transfer activity and mitochondrial dynamics should also be assessed because obesity may remodel contact sites for metabolic reasons that are not limited to Ca^2+^ transport. Ideally, contact distance or a defined tethering protein should be manipulated while the broader metabolic environment remains unchanged.

ER stress can further modify contact-site signaling by altering ER luminal Ca^2+^ content, IP_3_R activity, chaperone expression, and apoptotic pathways. Depending on the stage and severity of stress, the resulting mitochondrial phenotype may involve either insufficient physiological Ca^2+^ delivery or excessive transfer and matrix accumulation. In non-ovarian cardiac models, the IP_3_R1-GRP75-VDAC1 complex has been implicated in ER-stress-associated mitochondrial Ca^2+^ overload and oxidative injury (Yuan et al., 2022). These findings provide mechanistic support but should not be presented as direct ovarian evidence.

### 4.2 IP_3_R1 in porcine oocytes

Porcine oocytes provide direct reproductive evidence connecting IP_3_R1, mitochondrial Ca^2+^, and developmental outcomes. Reduced IP_3_R1 expression disturbed intracellular Ca^2+^ homeostasis, impaired cumulus expansion and polar-body extrusion, and reduced meiotic maturation. It also compromised cleavage after parthenogenetic activation. These changes were accompanied by ER and mitochondrial dysfunction, increased mitochondrial Ca^2+^ loading, oxidative stress, and apoptosis (Zhang et al., 2023).

A subsequent porcine study found that IP_3_R1 knockdown increased ER-mitochondria colocalization and reduced the distance between the organelles while altering pairwise associations within the IP_3_R1-GRP75-VDAC1 complex. IP_3_R1-GRP75 and IP_3_R1-VDAC1 associations were weakened, whereas the GRP75-VDAC1 association increased. These changes were accompanied by mitochondrial Ca^2+^ accumulation, increased ROS, reduced ATP production, impaired meiotic maturation and development after parthenogenetic activation, and increased apoptosis. Ruthenium red or N-acetylcysteine partially improved several outcomes (Zhang et al., 2024).

IP_3_R1 depletion paradoxically increased mitochondrial Ca^2+^ in the porcine studies (Zhang et al., 2024; Zhang et al., 2023), although reduced ER Ca^2+^ release might initially be expected to lower mitochondrial uptake. Several non-exclusive mechanisms could account for this result: compensatory remodeling of ER stores or alternative release pathways; altered contact geometry that produces localized transfer not captured by bulk cytosolic measurements; or secondary changes in membrane potential, matrix pH, or NCLX-dependent efflux that increase Ca^2+^ retention. The observed accumulation therefore cannot yet be attributed to a defined compensatory increase in ER-to-mitochondria Ca^2+^ transfer.

Time-resolved experiments are needed to distinguish the initiating defect from later consequences. Acute IP_3_R1 manipulation should be combined with simultaneous measurements of ER Ca^2+^ release, cytosolic transients, mitochondrial Ca^2+^ uptake and recovery, membrane potential, and ATP production. Rescue of IP_3_R1 expression or selective restoration of contact-site organization would provide stronger causal evidence than partial rescue with a general antioxidant or Ca^2+^-modifying drug.

IP_3_R1 signaling also intersects with cellular energy-sensing pathways. In porcine oocytes, pharmacological inhibition, siRNA-mediated depletion, and transcriptomic analyses supported a provisional model in which IP_3_R1/Ca^2+^ signaling engages CaMKK2 and the AMPK-mTOR-eIF4E axis to influence mitochondrial function and the oocyte-to-embryo transition (Teng et al., 2026). Because the study did not directly perturb or rescue each downstream node, the proposed pathway remains mechanistically suggestive rather than definitively established.

## 5 Mitochondrial Ca^2+^ in follicular cells and ovarian aging

Follicular somatic cells regulate the metabolic, hormonal, and ionic environment in which the oocyte develops. Granulosa cells provide metabolites and growth signals, participate in steroidogenesis, and communicate with the oocyte through differentiated cumulus cells. Their mitochondrial function can therefore influence oocyte developmental competence even when mitochondrial Ca^2+^ transport within the oocyte itself remains intact (Del Bianco et al., 2024).

Ca^2+^ signaling in granulosa cells regulates proliferation, differentiation, steroidogenic responses, apoptosis, and follicular atresia. In this compartment, mitochondrial Ca^2+^ dysfunction has been observed in both directions: insufficient uptake can impair energy metabolism and steroidogenesis, whereas excessive or sustained uptake can amplify oxidative stress and apoptosis. Toxicant, cystic-follicle, and genetic models provide examples of these context-dependent effects (Zhu et al., 2024; Zhang et al., 2025; Chen et al., 2026).

### 5.1 Cadmium and oxidative injury

Cadmium is an environmental and occupational toxicant associated with female reproductive dysfunction. In human KGN granulosa-like tumour cells, cadmium exposure increased intracellular free Ca^2+^, ROS production, and apoptosis while reducing mitochondrial membrane potential and ATP production (Xu G. et al., 2021). Because mitochondrial matrix Ca^2+^ was not directly quantified and MCU was not specifically manipulated, this study supports an association between disrupted cellular Ca^2+^ handling and mitochondrial dysfunction but does not directly demonstrate mitochondrial Ca^2+^ overload.

KGN cells are transformed and do not reproduce all properties of primary granulosa cells within intact follicles. Their proliferative state, steroidogenic phenotype, mitochondrial metabolism, and stress responses may differ from those of primary human granulosa cells. Cadmium also binds multiple cellular proteins and directly disrupts antioxidant and redox systems. The observed cytosolic Ca^2+^ elevation could therefore represent an initiating signal, an amplification mechanism, or a consequence of generalized cellular injury.

Avian studies provide more direct transporter-related evidence. In chicken granulosa cells, cadmium activated an IP_3_R-MCU-associated pathway, caused mitochondrial Ca^2+^ overload, reduced mitochondrial membrane potential, increased mitochondrial ROS, and promoted apoptosis. Ca^2+^ chelation or pharmacological MCU inhibition attenuated several of these changes. Furthermore, miR-129-1-3p directly targeted MCU and reduced cadmium-induced mitochondrial damage and apoptosis (Zhu et al., 2024).

In granulosa cells from laying hens exposed to H_2_O_2_, miR-129-1-3p similarly reduced MCU-associated mitochondrial Ca^2+^ signaling and attenuated autophagy-dependent cell death (Zhu et al., 2023). Together, these studies identify MCU as a potential amplification point in avian granulosa-cell injury. Nevertheless, genetic manipulation in primary mammalian granulosa cells and *in-vivo* reproductive assessment will be required before this mechanism can be generalized across species.

### 5.2 ER stress and granulosa-cell dysfunction

In porcine granulosa cells, dihydroartemisinin exposure increased intracellular and mitochondrial Ca^2+^ and activated PERK-eIF2α-ATF4-associated ER-stress signaling (Luo et al., 2020). These findings implicate Ca^2+^ dyshomeostasis in granulosa-cell injury without identifying a specific mitochondrial Ca^2+^ transporter. A separate porcine oocyte study reported impaired maturation after exposure to the same compound (Luo et al., 2018).

HB-EGF-associated estrogen hypersecretion and mitochondrial dysfunction have been described in a PCOS-related granulosa-cell model. HB-EGF increased cytosolic Ca^2+^ and induced loss of ATP, mtDNA copy number, and mitochondrial membrane potential, but mitochondrial matrix Ca^2+^ was not directly measured (Huang et al., 2022).

By contrast, in a PCOS mouse and granulosa-cell model, puerarin reduced cytosolic Ca^2+^ accumulation by restricting RyR- and IP_3_R-associated Ca^2+^ release. The study identified Mcu as a downstream target of NFATc, linked this pathway to mitochondrial Ca^2+^ uptake, and reported improvements in ATP content, mitochondrial membrane potential, and mitochondrial permeability transition (Wang et al., 2024). Nevertheless, puerarin affects multiple signaling pathways and should not be described as a selective MCU modulator.

Porcine cystic follicles were characterized by reduced MCU expression in granulosa cells. Mcu knockdown in normal granulosa cells impaired mitochondrial Ca^2+^ uptake, decreased mitochondrial membrane potential and ATP production, suppressed steroidogenic-gene expression and estradiol secretion, reduced AKT phosphorylation, and increased apoptosis (Chen et al., 2026). These findings support insufficient MCU-dependent Ca^2+^ uptake, rather than mitochondrial Ca^2+^ overload, as a contributor to granulosa-cell dysfunction in this model.

Granulosa-cell-specific *Foxj2* overexpression in mice upregulated MCU, induced mitochondrial Ca^2+^ overload and granulosa-cell apoptosis, increased follicular atresia, and produced a premature-ovarian-insufficiency-like phenotype (Zhang et al., 2025). This study provides direct evidence connecting an upstream transcriptional regulator with MCU-dependent mitochondrial Ca^2+^ dysregulation, although FOXJ2 overexpression may affect additional pathways beyond MCU.

In goat granulosa cells, neuromedin B binding to NMBR activated PLCβ1-dependent ER Ca^2+^ release and promoted IRE1α-IP_3_R-VDAC1-associated MAM formation. This response enhanced mitochondrial Ca^2+^ transfer, mitochondrial membrane potential, respiratory-chain activity, ATP production, mitochondrial fusion, and granulosa-cell proliferation (Xia et al., 2025). These findings provide contact-site and mitochondrial Ca^2+^ evidence in a caprine model but should not be presented as human ovarian evidence.

### 5.3 Cumulus-oocyte metabolic coupling

Cumulus cells and the oocyte form a metabolically integrated unit. Cumulus cells convert glucose into substrates such as pyruvate that can be oxidized by the oocyte, and transfer amino acids, nucleotides, ions, and signaling molecules through gap junctions. The oocyte, in turn, regulates cumulus-cell differentiation and metabolism through paracrine factors (Del Bianco et al., 2024). Mitochondrial Ca^2+^ in cumulus cells could influence oocyte competence through several mechanisms. Insufficient physiological uptake may limit ATP production and substrate processing, whereas excessive uptake may promote ROS generation and apoptosis, impair gap-junction communication, or alter the composition of follicular fluid.

Direct Ca^2+^-imaging data in cumulus cells are sparse. Most available values come from bulk cytosolic indicators in cumulus-oocyte complexes (COCs), and calibrated mitochondrial Ca^2+^ measurements specifically in cumulus cells remain limited. The cumulus-cell side of the electro-metabolic coupling is therefore inferred from indirect readouts (ATP, mtDNA copy number, mitochondrial membrane potential) rather than from calibrated mitochondrial Ca^2+^ fluxes. Until cumulus-side calibration becomes routine, conclusions about cumulus-cell mitochondrial Ca^2+^ should be regarded as inferential.

Gap-junction-mediated coupling permits ions and signaling molecules to pass between cumulus cells and the oocyte, providing a plausible route by which a cumulus-cell Ca^2+^ disturbance could alter oocyte competence (Del Bianco et al., 2024). However, no study has yet reported a cumulus-cell-specific conditional knockout of MCU, MICU1, MICU2, or NCLX. Cumulus-cell mitochondrial Ca^2+^ cannot therefore be assumed to represent that of the corresponding oocyte, and metabolic coupling does not guarantee identical transporter expression, membrane potential, Ca^2+^ dynamics, or stress responses.

### 5.4 Chronological ovarian aging

Chronological ovarian aging involves progressive follicle depletion together with deterioration in oocyte quality. Reported mitochondrial abnormalities include altered morphology and distribution, reduced respiratory performance, increased ROS, mtDNA instability, impaired mitochondrial quality control, and disturbed communication among intracellular organelles (Bahety et al., 2024; Yildirim and Seli, 2024a; Nagaraju et al., 2026).

Aging may narrow the functional window for mitochondrial Ca^2+^ signaling by reducing physiological uptake when membrane potential declines, while altered ER-mitochondria coupling, efflux, or matrix buffering may promote local retention. Reduced antioxidant capacity could further increase oxidative injury at a given Ca^2+^ exposure. These mechanisms are biologically plausible, but direct, stage-resolved, and quantitatively calibrated evidence from human oocytes remains limited.

Candidate diagnostic genes for human oocyte aging have been identified through transcriptomic analysis and machine-learning approaches. PDIK1L, SIRT1, and MCU were identified as hub genes in one such analysis (Luo et al., 2025). These findings can prioritize pathways for functional investigation but cannot establish mitochondrial Ca^2+^ concentration, uptake, efflux, or recovery kinetics. Validation will require donated human oocytes characterized by maternal age, ovarian diagnosis, stimulation protocol, maturation stage, and culture conditions.

### 5.5 Postovulatory aging

Postovulatory aging occurs after ovulation or prolonged *in-vitro* culture and is distinct from long-term reproductive senescence. It is associated with spindle abnormalities, cortical changes, oxidative stress, mitochondrial dysfunction, altered Ca^2+^ signaling, reduced fertilization competence, and impaired embryo development (Liu et al., 2024; Ma et al., 2024).

Salidroside improved mitochondrial distribution, mitochondrial membrane potential, ATP content, redox balance, spindle organization, and developmental competence in postovulatory-aged mouse oocytes. It also restored cytosolic and mitochondrial Ca^2+^ indicators toward control levels (Liu et al., 2024).

Petunidin-3-O-(6-O-p-coumaroyl)-rutinoside-5-O-glucoside similarly improved mitochondrial distribution, membrane potential, ATP production, oxidative balance, and developmental competence in aged mouse oocytes. Cytosolic and mitochondrial Ca^2+^ indicators were also restored, and transcriptomic analyses implicated the putrescine pathway (Ma et al., 2024).

These studies directly included mitochondrial Ca^2+^ measurements, but the tested compounds are pleiotropic and the measurements were primarily endpoint fluorescence signals rather than calibrated Ca^2+^ flux analyses. The results therefore do not establish MCU, MICU1/2, or NCLX as the direct therapeutic target. Transporter-specific perturbation and quantitative measurements of uptake and recovery kinetics are required before these compounds can be described as direct regulators of mitochondrial Ca^2+^ transport.

### 5.6 Avian ovarian aging

Aging laying hens provide a model of progressive follicular decline and granulosa-cell dysfunction. In naturally aged laying hens and a D-galactose-induced avian follicular-cell senescence model, apigenin improved ovarian or follicular phenotypes, modulated the expression of several Ca^2+^-handling genes, and attenuated ER-stress signaling (Gao et al., 2026).

However, the study did not provide calibrated measurements of mitochondrial Ca^2+^ flux or demonstrate direct regulation of MCU, MICU1/2, or NCLX. Moreover, avian follicular organization, ovulation patterns, and reproductive physiology differ substantially from those of mammals. These findings may reveal conserved Ca^2+^-ER-stress pathways but should not be translated directly into human ovarian treatment.

### 5.7 Cell-specific functions of mitochondrial Ca^2+^ within the ovarian follicle

This review focuses on four principal follicular cell populations: the oocyte, mural granulosa cells, cumulus cells, and theca cells. Each occupies a different position in the electro-metabolic circuit and may therefore use mitochondrial Ca^2+^ differently.

Mural granulosa cells support follicular metabolism, signaling, and steroidogenesis, as illustrated by the models discussed in Sections 5.1 and 5.2. Theca cells are the principal ovarian source of LH-stimulated androgen production and rely on mitochondrial cholesterol import and steroidogenic enzymes. However, evidence linking mitochondrial Ca^2+^ directly to steroid output comes mainly from adrenal and Leydig-cell systems (Stocco, 2001; Lalevée et al., 2003; Hales et al., 2005); whether mitochondrial Ca^2+^ flux is rate-limiting in primary theca cells remains unknown.

Cumulus cells are predominantly glycolytic and supply metabolic substrates and signaling molecules to the oocyte through gap-junction-mediated coupling (Del Bianco et al., 2024). The oocyte uses these substrates for mitochondrial oxidative metabolism, while the ER serves as its major intracellular Ca^2+^ store. As discussed in Section 5.3, calibrated cumulus-cell mitochondrial Ca^2+^ measurements remain limited.

Mitochondrial Ca^2+^ should consequently be understood as a cell-type-specific, dynamic balance among uptake, buffering, metabolic utilization, and efflux. A finding in 1 cell type cannot be transferred to another without empirical support.

### 5.8 Folliculogenesis and steroidogenesis: Mitochondrial Ca^2+^ coupling

Folliculogenesis depends on coordinated proliferation, differentiation, angiogenesis, steroidogenesis, and oocyte support. Mitochondrial Ca^2+^ can influence these processes indirectly through dehydrogenase activation, ATP supply, redox balance, and apoptosis, while granulosa, cumulus, and theca cells occupy different positions in the follicular circuit. The available ovarian studies therefore support a coupling hypothesis rather than a single follicle-wide Ca^2+^ phenotype.

At the mitochondrial step of steroidogenesis, StAR supports cholesterol transfer across the mitochondrial membranes (Stocco, 2001). Evidence that cytosolic-to-mitochondrial Ca^2+^ transfer modulates steroid output is well established in adrenal and Leydig-cell systems (Lalevée et al., 2003; Hales et al., 2005), but these mechanisms cannot be assumed to operate identically in ovarian cells. Within an ovarian model, Mcu knockdown in porcine granulosa cells reduced mitochondrial Ca^2+^ uptake, membrane potential, ATP production, steroidogenic-gene expression, and estradiol secretion while increasing apoptosis (Chen et al., 2026). This provides direct transporter-perturbation evidence in a non-human ovarian cell, although energetic failure and loss of viability may contribute to the steroidogenic phenotype. Equivalent transporter-resolved evidence remains unavailable in primary human granulosa or theca cells. Mitochondrial Ca^2+^ should therefore be considered a plausible regulator of ovarian steroidogenesis, not an established rate-limiting or therapeutically selective target.

### 5.9 Human reproductive tissue: Current evidence and limits

Direct mitochondrial Ca^2+^ measurement in viable human oocytes is constrained by tissue availability, ethical review, invasiveness, and selection biases in oocytes not used clinically. Most functional studies are performed in mouse or porcine oocytes, with avian, zebrafish, *Xenopus*, and sea-urchin models providing complementary information. Available human evidence derives largely from transcriptomic analyses, observational mitochondrial measurements, cumulus cells, or oocytes unsuitable for clinical use (Yildirim and Seli, 2024a; Luo et al., 2025). Direct, quantitatively calibrated measurements of mitochondrial Ca^2+^ dynamics in viable human oocytes remain scarce.

Commonly studied human sources include (i) immature germinal-vesicle-stage oocytes from stimulated cycles that fail to mature *in vitro*; (ii) post-mature or failed-to-fertilize metaphase-II oocytes after intracytoplasmic sperm injection; and (iii) ovarian tissue removed for clinical indications and used under approved consent. Each source is selected rather than physiologically neutral. Failed-to-fertilize oocytes may carry activation, chromosomal, or cytosolic defects; GV oocytes do not represent MII-stage physiology; and ovarian tissue obtained for a clinical indication may reflect the underlying disease or treatment exposure.

Cumulus cells are routinely removed during assisted reproduction and represent accessible human material. Their molecular profiles have been associated with oocyte competence and embryo outcome and can provide a window into follicular metabolism (Del Bianco et al., 2024; Yildirim and Seli, 2024a). However, cumulus-cell mitochondrial Ca^2+^ cannot currently be assumed to represent the corresponding oocyte. Paired cumulus-oocyte studies are required, and biomarker measurement should not compromise the developmental use of the oocyte.

The link between mitochondrial Ca^2+^ and ovarian steroidogenesis remains to be established. StAR mediates cholesterol transfer from the outer to the inner mitochondrial membrane (Stocco, 2001), and cytosolic-to-mitochondrial Ca^2+^ transfer has been linked to steroidogenesis in adrenal and Leydig-cell models (Lalevée et al., 2003; Hales et al., 2005). These findings cannot be assumed to apply directly to ovarian cells: no study has simultaneously manipulated a mitochondrial Ca^2+^ transporter and measured steroid output in primary human ovarian cells.

Two practical constraints apply to any human mitochondrial Ca^2+^ study. First, probe loading or microinjection is invasive and incompatible with embryos intended for transfer. Second, sample sizes are small and clustering by donor must be accounted for in study design and statistical analysis (see Section 8.5). Multiple oocytes from the same donor are not independent biological replicates. The conclusions that can be drawn from human material are therefore necessarily narrower than those available from animal models, and a candidate biomarker must show predictive value beyond maternal age, ovarian diagnosis, oocyte morphology, embryo morphokinetics, and ploidy where available (Yildirim and Seli, 2024a).

### 5.10 Pathological contexts matrix

**FIGURE 5:**
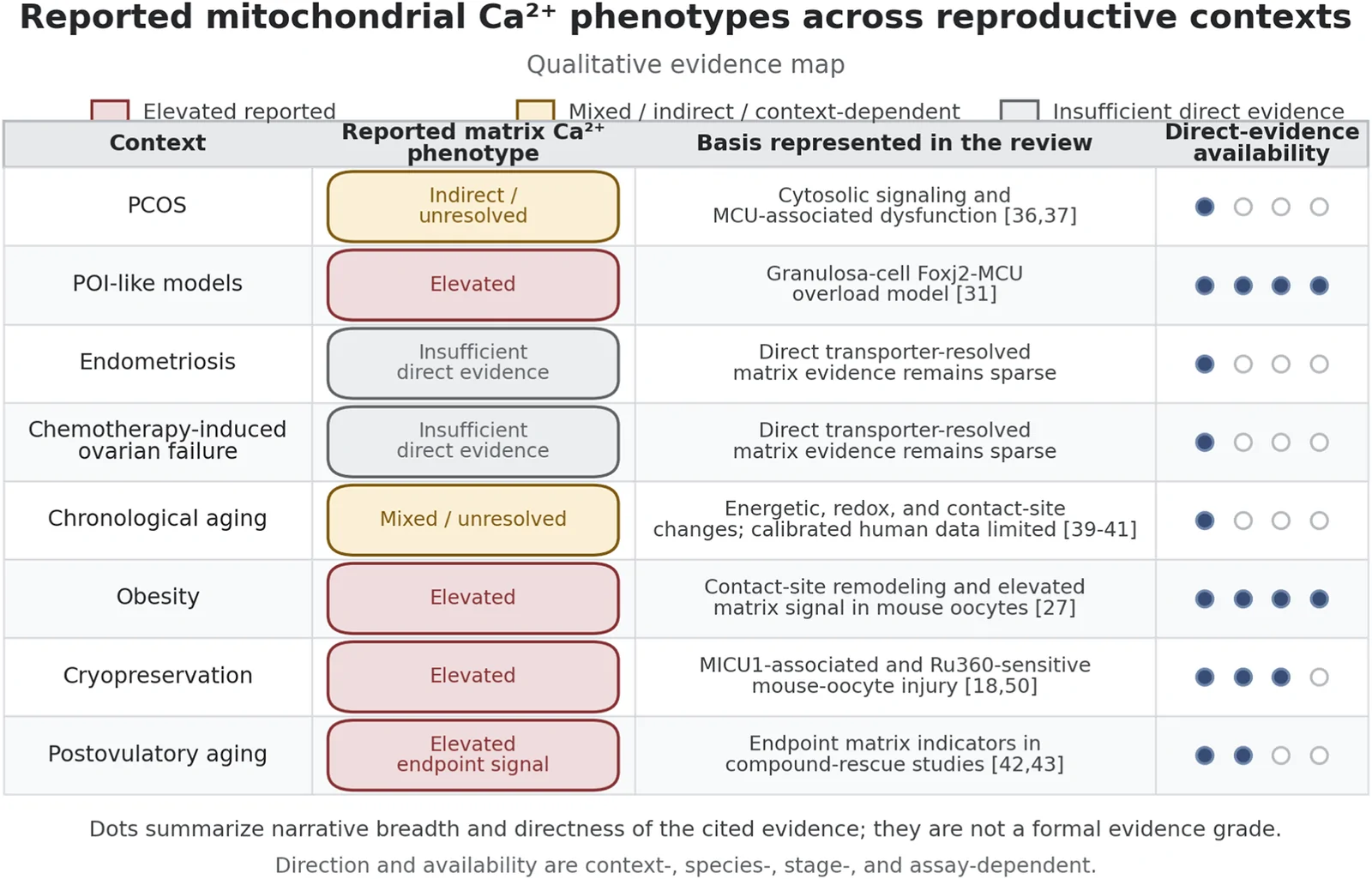
Qualitative summary of reported mitochondrial Ca^2+^ phenotypes across ovarian and environmental contexts. Colors distinguish reported phenotype direction and evidentiary qualification; dots reflect the narrative breadth and directness of available evidence, not a formal evidence grade. Grey denotes insufficient direct mitochondrial Ca^2+^ evidence. Abbreviations: PCOS, polycystic ovary syndrome; POI, premature ovarian insufficiency.

The available evidence does not support a single mitochondrial Ca^2+^ phenotype across ovarian disorders. PCOS and premature-ovarian-insufficiency models include both altered cytosolic Ca^2+^ signaling and MCU-associated mitochondrial dysfunction (Zhang et al., 2025; Huang et al., 2022; Wang et al., 2024), whereas obesity, chronological aging, cryopreservation, and postovulatory aging involve distinct combinations of contact-site remodeling, energetic impairment, oxidative stress, and Ca^2+^ dysregulation (Zhao et al., 2017; Yildirim and Seli, 2024a; Liu et al., 2024; Ma et al., 2024; Lan et al., 2022; Sun et al., 2023). Direct transporter-resolved evidence remains sparse for endometriosis and chemotherapy-induced ovarian failure. Figure 5 therefore summarizes both the reported direction of mitochondrial Ca^2+^ change and the relative availability of direct evidence, rather than treating these conditions as a single overload syndrome.

## 6 Mitochondrial Ca^2+^ in oocyte meiotic maturation

Oocyte maturation comprises germinal-vesicle breakdown (GVBD), chromosome condensation, spindle assembly and migration, asymmetric cytokinesis, and first-polar-body extrusion. Mitochondria redistribute around the germinal vesicle, chromosomes, and meiotic spindle as these processes create changing local energy demands (Bahety et al., 2024; Wang et al., 2020). Mitochondrial Ca^2+^ can couple those demands to ATP production, but the available mouse-oocyte evidence supports a functional window: insufficient entry impairs bioenergetics and meiotic progression, whereas excessive matrix loading promotes oxidative stress and delays maturation (Zhang et al., 2020; Zhang et al., 2021).

This bidirectional model does not imply fixed Ca^2+^ thresholds across species or stages. Matrix loading depends on cytosolic Ca^2+^, membrane potential, MCU-complex gating, efflux, buffering, and exposure duration. Intervention effects must therefore be interpreted against baseline Ca^2+^ status and the developmental context rather than classified as uniformly protective or harmful.

### 6.1 Requirement for MCU-dependent uptake

In mouse oocytes, siRNA-mediated Mcu knockdown reduced GVBD and metaphase-II progression, increased spindle abnormalities, lowered mitochondrial Ca^2+^ and ATP, and altered mitochondrial mass and membrane potential. Ru360 reproduced the reductions in mitochondrial Ca^2+^ and ATP and produced similar meiotic and spindle phenotypes, although the complete mitochondrial and signaling analyses were performed primarily in the knockdown arm (Zhang et al., 2021).

Mcu knockdown also increased and redistributed phosphorylated AMPK, and AMPK inhibition with Compound C partially restored GVBD and first-polar-body extrusion (Zhang et al., 2021). These results connect reduced mitochondrial Ca^2+^ entry to energetic stress and AMPK-dependent meiotic control, but they do not establish ATP depletion as the sole cause of the phenotype because MCU loss can also alter redox signaling, mitochondrial organization, local Ca^2+^ buffering, and spindle regulation.

Mechanistic separation will require complementary rescue experiments. Restoring ATP without restoring MCU conductance would test whether energetic failure is sufficient to explain the maturation defect, whereas an RNAi-resistant MCU construct could test whether channel function rescues it. Acute or inducible manipulation would further reduce the compensatory changes in mitochondrial biogenesis and Ca^2+^-handling pathways that may accompany prolonged knockdown.

### 6.2 Excess mitochondrial Ca^2+^ and meiotic injury

Evidence for the opposite limb of the functional window comes from mouse oocytes in which mitochondrial Ca^2+^ was increased by Micu1/2 or Nclx knockdown. Elevated matrix Ca^2+^ was associated with increased ROS, loss of mitochondrial membrane potential, ATP disturbance, and delayed meiotic progression. Reducing Ca^2+^ entry with Ru360 or Mcu knockdown attenuated several of these abnormalities. In oocytes from high-fat-diet mice, the same entry-limiting interventions improved mitochondrial and maturation outcomes (Zhang et al., 2020).

These results should not be generalized to H_2_O_2_- or cadmium-exposed granulosa cells, which are different cellular models (Zhu et al., 2024; Xu G. et al., 2021; Zhu et al., 2023), or treated as evidence that MCU inhibition benefits healthy oocytes. Together with the loss-of-uptake findings in unstressed oocytes (Zhang et al., 2021), they instead show that the direction of benefit depends on the initial matrix Ca^2+^ state and the experimental injury.

### 6.3 Spatial heterogeneity of mitochondrial Ca^2+^

Mt-GCaMP6s imaging in mouse oocytes showed higher mitochondrial Ca^2+^ around the germinal vesicle than at the cortex during maturation; after GVBD, mitochondria with higher Ca^2+^ were enriched around chromosomes and the spindle. Cytosolic and mitochondrial Ca^2+^ changes were synchronous during parthenogenetic activation (Wang et al., 2020). These observations support spatial heterogeneity in mouse oocytes, but they do not demonstrate the same pattern in porcine oocytes or prove that experimentally disrupting the pattern impairs fertilization-associated Ca^2+^-oscillation decoding.

### 6.4 Interaction with plasma-membrane and ER Ca^2+^ pathways

Transport upstream of the mitochondrion changes the Ca^2+^ available for matrix uptake. Oocyte-specific PMCA1 deletion prolonged total cytosolic Ca^2+^ exposure after fertilization and altered offspring growth (Savy et al., 2022). IP_3_R1 effects were context dependent: its downregulation decreased mitochondrial Ca^2+^ and apoptosis in oocytes from obese mice (Zhao et al., 2017), whereas siRNA-mediated depletion increased mitochondrial Ca^2+^ accumulation and oxidative injury in porcine oocytes (Zhang et al., 2024; Zhang et al., 2023). Thus, IP_3_R1 perturbation cannot be assigned a uniform effect across species, metabolic states, and experimental designs.

### 6.5 AMPK and mTOR signaling across the oocyte-to-embryo transition

In mouse oocytes, reduced MCU-dependent Ca^2+^ entry lowered ATP, increased phosphorylated AMPK, and impaired meiotic progression, with partial rescue after AMPK inhibition (Zhang et al., 2021). A porcine study linked IP_3_R1/Ca^2+^-CaMKK2 signaling to the AMPK-mTOR-eIF4E axis, mitochondrial function, and development during *in-vitro* maturation and especially the post-activation oocyte-to-embryo transition (Teng et al., 2026). The latter evidence should not be interpreted as showing that mitochondrial Ca^2+^ alone determines meiotic resumption; it extends the metabolic-signaling framework into egg activation and early development.

### 6.6 Obesity and maternal-diet effects across developmental stages

Oocytes from obese mice showed increased ER-mitochondria association, mitochondrial Ca^2+^, apoptosis, and impaired cytoplasmic maturation; reducing IP_3_R1, PACS-2, or MCU-dependent entry alleviated selected defects (Zhang et al., 2020; Zhao et al., 2017). These findings support mitochondrial Ca^2+^ excess in this obesity model but do not establish obesity as a uniform overload state in all species or patients.

Other dietary studies concern different developmental stages. Maternal omega-3 supplementation altered active mitochondrial distribution, mitochondrial Ca^2+^, and ROS in ovulated mouse oocytes, with developmental effects that differed between *in-vitro* fertilization and culture of in-vivo-derived zygotes (Wakefield et al., 2008). Maternal high- and low-protein diets reduced mitochondrial membrane potential and increased mitochondrial Ca^2+^ in two-cell embryos (Mitchell et al., 2009). These embryo-stage observations should not be presented as direct evidence about meiotic maturation. Because substrate and ionic composition, oxygen tension, and pH can modify cellular bioenergetics and Ca^2+^ handling, studies should report complete maturation and culture conditions.

### 6.7 Cryopreservation

Vitrification can disturb mitochondrial function, ER organization, spindle integrity, and Ca^2+^ homeostasis, but results differ with oocyte stage and intervention (Lan et al., 2022; Sun et al., 2023). In vitrified-thawed mouse metaphase-II oocytes, MICU1 abundance and mitochondrial Ca^2+^ increased while membrane potential and ATP decreased. Post-warming treatment with DS16570511, designated by the study as a MICU1 inhibitor, further reduced ATP, cleavage, and blastocyst development; MCU-i4, designated as a MICU1 activator, improved membrane potential and ATP without significantly improving cleavage or blastocyst rates (Lan et al., 2022).

The pharmacological classification and target specificity of these compounds require independent validation. The study therefore supports a provisional association between MICU1-linked uptake and energetic compensation after warming, rather than proving that increased mitochondrial Ca^2+^ is beneficial or that MICU1 is the only relevant target.

A separate study vitrified immature germinal-vesicle-stage mouse oocytes. Vitrification increased mitochondrial Ca^2+^ and impaired mitochondrial function, spindle organization, chromosome alignment, kinetochore-microtubule attachment, spindle-assembly-checkpoint function, and first-polar-body extrusion. Ru360 reduced mitochondrial Ca^2+^ and partially rescued these defects (Sun et al., 2023).

The MICU1 and Ru360 results are not directly contradictory because they used different maturation stages, pharmacological targets, and treatment windows. They indicate that an acute reduction in excessive loading may benefit injured germinal-vesicle-stage oocytes, whereas sustained restriction of uptake during post-warming recovery of metaphase-II oocytes may worsen ATP insufficiency. Neither study establishes an optimal therapeutic window or MCU-specific causality.

### 6.8 Toxicant-associated meiotic injury and causal limits

In oxybenzone-exposed mouse oocytes, melatonin reduced endpoint Fluo-4 and Rhod-2 fluorescence and improved mitochondrial dynamics, membrane potential, redox balance, and spindle organization (Sun et al., 2023). Because melatonin is pleiotropic and these dyes did not provide calibrated mitochondrial Ca^2+^ flux, the rescue cannot be assigned specifically to MCU or interpreted as direct normalization of mitochondrial Ca^2+^ transport.

A causal overload model requires the mitochondrial Ca^2+^ rise to precede ROS accumulation, depolarization, spindle disruption, and apoptosis, followed by selective rescue when matrix loading is prevented. Conversely, a proposed uptake-deficiency mechanism requires restoration of entry to rescue bioenergetics and maturation. This complete temporal and compartment-resolved evidential sequence has not yet been implemented in most reproductive injury models.

## 7 Fertilization, Ca^2+^ oscillations, and early embryonic development

Mammalian egg activation is initiated principally by sperm-borne PLCζ. After gamete fusion, PLCζ enters the ooplasm and hydrolyses phosphatidylinositol 4,5-bisphosphate to generate IP_3_, which activates IP_3_R1 in the ER and initiates a prolonged series of cytosolic Ca^2+^ oscillations (Swann, 2023; Swann, 2025).

The number, amplitude, frequency, and duration of these oscillations influence cortical-granule exocytosis, exit from meiotic arrest, pronuclear formation, and subsequent development (Swann, 2023; Swann, 2025). Egg activation is therefore encoded by a temporal Ca^2+^ pattern rather than by a single elevation, an important distinction when comparing fertilization with artificial activation.

Mitochondria support this process primarily by coupling Ca^2+^ signaling to ATP production. Mitochondrial ATP can modulate IP_3_R1 sensitivity and sustain ATP-dependent Ca^2+^ clearance and ER refilling (Swann, 2023; Swann, 2025; Ikie-Eshalomi et al., 2023). Direct matrix Ca^2+^ uptake may also shape local signaling, but mammalian evidence for this role is less complete than the evidence for cytosolic oscillations and metabolic activation.

### 7.1 Fertilization-associated mitochondrial Ca^2+^ uptake and ATP production

In sea-urchin eggs, fertilization increased mitochondrial Ca^2+^ uptake and expanded the mitochondrial Ca^2+^ pool, consistent with a temporary sink during egg activation (Girard et al., 1991). Mammalian eggs differ because repetitive cytosolic oscillations persist for several hours while metabolism, organelle organization, and protein synthesis are changing (Swann, 2023; Swann, 2025). Results from sea urchins should therefore not be treated as direct measurements of mammalian matrix Ca^2+^ dynamics.

By analogy with established NCLX transport mechanisms and reproductive genetic evidence, NCLX-dependent recovery may determine whether successive uptake events remain discrete or accumulate as matrix load (Fan et al., 2025; Garbincius et al., 2025; Meng et al., 2023). However, no study has yet combined NCLX-specific manipulation with calibrated mitochondrial Ca^2+^ imaging through an intact mammalian fertilization-associated oscillatory train. Its proposed role in oscillation decoding therefore remains a mechanistic hypothesis.

ATP dynamics are not a simple readout of cytosolic Ca^2+^. In mouse eggs, sperm induced an initial ATP increase near the first Ca^2+^ transient and a distinct second increase approximately one hour later. PLCζ expression or thimerosal-induced oscillations caused smaller or slower ATP changes and did not reproduce the secondary rise (Ikie-Eshalomi et al., 2023).

When low concentrations of BAPTA suppressed most sperm-induced oscillations, the sperm still induced additional phases of ATP elevation (Ikie-Eshalomi et al., 2023). This finding supports a sperm-associated component of metabolic activation that is not reproduced by PLCζ-driven Ca^2+^ oscillations alone, although the responsible sperm factor and mechanism remain unresolved.

The production and use of ATP should consequently be considered separately. ATP supports SERCA- and PMCA-mediated Ca^2+^ clearance, cytoskeletal remodeling, exocytosis, pronuclear formation, phosphorylation, and protein synthesis; declining ATP may in turn modify later Ca^2+^ transients (Swann, 2023; Swann, 2025; Ikie-Eshalomi et al., 2023). This creates reciprocal coupling between mitochondrial metabolism and ER Ca^2+^ release without showing that every ATP change is caused by matrix Ca^2+^.

Resolving this coupling will require simultaneous, time-resolved measurement of cytosolic and mitochondrial Ca^2+^, ATP, mitochondrial membrane potential, and respiration. Separate endpoint assays cannot establish the temporal order needed for a causal model.

### 7.2 Artificial activation and pharmacological evidence

The Ca^2+^ ionophore A23187 has been used to activate human oocytes after failed ICSI, and usable blastocysts were obtained in a clinical rescue study (Xu Z. et al., 2021). This demonstrates that an imposed Ca^2+^ rise can initiate development in selected activation-failure cases; it does not show that ionophore exposure reproduces physiological PLCζ-driven oscillations or establishes long-term safety.

In strontium-activated mouse oocytes, CGP37157 reduced survival, Ca^2+^ oscillations, mitochondrial activity, pronuclear formation, and two-cell development. Erastin also altered mitochondrial and Ca^2+^ responses and reduced pronuclear formation (Wang et al., 2021b). These results associate mitochondrial Ca^2+^ handling with artificial activation, but CGP37157 affects targets beyond NCLX and erastin is a pleiotropic ferroptosis inducer rather than a selective VDAC probe.

Human ionophore rescue and mouse strontium activation answer different questions and should not be combined as evidence for a single therapeutic mechanism. Neither model reproduces sperm-triggered signaling in full, and the pharmacological experiments do not establish transporter-specific causality.

### 7.3 Genetic and ER-upstream control of the oocyte-to-embryo transition

As detailed in Section 3.3, maternal NLRP14 stabilizes NCLX through regulation of K27-linked ubiquitination. Maternal Nlrp14 deficiency reduced NCLX abundance, disrupted mitochondrial Ca^2+^ homeostasis and morphology, and blocked development before the two-cell stage. Exogenous Nclx mRNA reduced embryonic mortality but did not restore two-cell development (Meng et al., 2023). The incomplete rescue indicates that NCLX is an important component of a broader NLRP14-dependent maternal cytoplasmic program.

Porcine studies also connect IP_3_R1-dependent ER Ca^2+^ signaling with maturation, pronuclear formation, and early development (Zhang et al., 2024; Zhang et al., 2023; Teng et al., 2026). One proposed pathway links IP_3_R1/Ca^2+^-CaMKK2 signaling to AMPK-mTOR-eIF4E activity and mitochondrial metabolism during the oocyte-to-embryo transition (Teng et al., 2026). Because IP_3_R1 perturbation has produced context-dependent mitochondrial Ca^2+^ responses and these studies use related porcine systems, the pathway is supported but not yet independently established as a uniform causal sequence.

### 7.4 Bidirectional mitochondrial Ca^2+^ disruption and early development

In mouse metaphase-II oocytes, spermine increased mitochondrial Ca^2+^ and phosphorylated MAPK/ERK, whereas Ru360 decreased mitochondrial Ca^2+^ and phosphorylated MAPK/ERK. Both interventions reduced mitochondrial membrane potential and ATP, increased spindle or chromosomal abnormalities, and impaired pronuclear formation and preimplantation development after parthenogenetic activation (Zhang et al., 2022). Thus, opposite Ca^2+^ perturbations produced opposite MAPK responses but converged on developmental impairment.

In pigs, Rhod-2 fluorescence was higher in presumptive zygotes six hours after IVF than in mature oocytes, not parthenogenetic controls. Ruthenium red reduced Rhod-2 fluorescence and MICU1 abundance; 20 μM ruthenium red improved blastocyst development, whereas 10 μM did not and 40 μM produced no improvement (Jegal et al., 2020). These findings demonstrate a dose-dependent association, not that the physiological post-fertilization Ca^2+^ rise is intrinsically pathological.

The mouse and porcine studies are consistent with a functional range but rely on spermine, Ru360, or ruthenium red. Because these compounds have broader actions and Rhod-2 is not a calibrated matrix-flux measurement, the experiments remain pharmacological evidence rather than definitive proof of MCU-specific mechanisms.

### 7.5 Culture environment and long-term developmental consequences

Fertilization media differ in ionic ratios and energy substrates that can alter Ca^2+^ excitability and metabolic recovery. Although framed around IVF media, a mouse study used ICSI and simultaneously recorded cytosolic Ca^2+^ with Fura-2 and mitochondrial redox responses with FAD autofluorescence. Medium composition altered PLCζ-dependent oscillatory profiles, metabolic responses, and developmental potential (Ozil et al., 2026). The study did not directly measure mitochondrial matrix Ca^2+^.

Oocyte-specific PMCA1 depletion prolonged cytosolic Ca^2+^ exposure after fertilization without significantly changing female litter size or time to first litter. Offspring growth was altered, and male offspring exposed to the abnormal fertilization-associated Ca^2+^ signal showed altered body composition (Savy et al., 2022). The study did not report male subfertility and did not measure mitochondrial Ca^2+^, so it cannot establish a matrix-Ca^2+^ mechanism.

Developmental assessment should therefore extend beyond pronuclear formation, cleavage, and blastocyst yield to chromosome segregation, implantation, pregnancy loss, placental development, fetal growth, postnatal metabolism, and fertility. Such outcomes are particularly important when Ca^2+^ handling is manipulated in the maternal germline.

Overall, cytosolic Ca^2+^ oscillations and ATP dynamics at fertilization are well supported, whereas direct, transporter-resolved measurements of mitochondrial Ca^2+^ during mammalian fertilization remain limited. Genetic models such as Nlrp14 deficiency strengthen the causal framework, but pharmacological and cross-species findings must remain separated by activation mode, developmental stage, and evidence strength. Table 2 summarizes the reproductive evidence and its principal limitations.

**TABLE 2:** Reproductive evidence.

Reproductive context	Model basis	Main finding	Principal limitation
Oocyte maturation	Mouse oocytes	MCU-dependent uptake supports meiotic progression and ATP production (Zhang et al., 2021)	Evidence is mainly from mouse in-vitro maturation experiments
Ca^2+^ overload	Mouse oocyte transporter-knockdown and obesity-associated models	Excess matrix Ca^2+^ increases ROS and delays meiosis (Zhang et al., 2020)	Transporter perturbation and metabolic-model effects are difficult to separate
Spatial signaling	Mouse oocytes	Perinuclear mitochondria show distinct Ca^2+^ dynamics (Wang et al., 2020)	Spatial correlation does not establish causality
Fertilization	Sea-urchin Ca^2+^-pool and mouse ATP studies	Mitochondria participate in Ca^2+^-metabolic coupling (Girard et al., 1991; Ikie-Eshalomi et al., 2023)	Cross-species findings differ, and ATP changes are not determined exclusively by Ca^2+^
Oocyte-to-embryo transition	Mouse maternal Nlrp14 model	NLRP14 stabilizes NCLX and supports early development (Meng et al., 2023)	NLRP14 also affects the broader maternal cytoplasm
IP_3_R1 signaling	Porcine oocytes and embryos	IP3R1 influences maturation, metabolism, and embryo development (Zhang et al., 2024; Zhang et al., 2023; Teng et al., 2026)	Cytosolic and mitochondrial consequences of IP3R1 perturbation are difficult to separate
Cryopreservation	Mouse MII- and GV-stage oocytes	MICU1-associated dysregulation and Ru360-sensitive injury occur after vitrification (Lan et al., 2022; Sun et al., 2023)	Stage, pharmacological specificity, and human relevance remain uncertain
Granulosa-cell stress	Chicken primary cells and the human KGN cell line	Cadmium and oxidative stress involve Ca^2+^-mitochondrial dysfunction; MCU-specific evidence is avian (Zhu et al., 2024; Xu et al., 2021a; Zhu et al., 2023)	The human cell-line study did not measure MCU or mitochondrial Ca^2+^ directly

## 8 Experimental approaches and methodological limitations

Studies of mitochondrial Ca^2+^ use organelle-targeted indicators, genetic perturbation, biochemical uptake assays, electrophysiology, and parallel measurements of mitochondrial function. These approaches interrogate different quantities: matrix concentration, influx and efflux rates, buffering, spatial distribution, and integrated exposure. Agreement among them strengthens interpretation, but none can be substituted automatically for another.

Fertilization-associated Ca^2+^ oscillations illustrate the need for measurement discipline. In mouse eggs, the first transient begins within approximately one minute of gamete fusion, followed typically by 10–20 transients separated by about 10–20 min over 3–4 h (Swann, 2023; Swann, 2025). Absolute peak concentrations are less certain because many recordings were not calibrated; a Mag-Fura-2 study cited in the recent review estimated peaks of approximately 1–3 μM (Swann, 2025). Oscillation patterns differ among species, so numerical ranges should be reported with the species, probe, loading method, calibration procedure, and sampling rate rather than presented as universal constants.

### 8.1 Genetically encoded and synthetic Ca^2+^ indicators

CEPIA variants enable genetically encoded Ca^2+^ imaging in the ER, mitochondrial matrix, and cytosol. Their spatiotemporal resolution can resolve heterogeneous uptake among individual mitochondria while organellar and cytosolic signals are measured in parallel (Suzuki et al., 2014).

R-CEPIA3mt and R-CEPIA4mt were engineered with Ca^2+^ affinities suited to mitochondrial measurements and can be combined with compatible green indicators or optogenetic tools (Kanemaru et al., 2020). Probe selection must nevertheless be matched to the expected concentration range; a sensor operating near saturation cannot resolve further increases.

Ratiometric and bioluminescent indicators have enabled mitochondrial Ca^2+^ imaging in intact zebrafish embryos, including during spontaneous skeletal-muscle contraction (Mizuno et al., 2013; Vicente et al., 2019). These studies demonstrate *in-vivo* feasibility but do not validate the same expression, targeting, kinetics, or buffering behavior in mammalian oocytes and preimplantation embryos.

Split-MEGIC reconstitutes fluorescence at mitochondria-ER junctions and can report local Ca^2+^ activity (Olszakier et al., 2025). Its junctional applications have been developed mainly in neural systems, including dendritic spines and larval zebrafish neurons. Expression, targeting, reconstitution efficiency, and effects on contact-site organization therefore require independent validation in reproductive cells.

Genetically encoded indicators can perturb the process being measured. High expression or affinity may buffer Ca^2+^, reduce apparent peak amplitude, or prolong recovery, while variable expression and targeting efficiency can create differences among oocytes or mitochondrial subpopulations (Suzuki et al., 2014; Kanemaru et al., 2020). Expression should be titrated, and developmental competence should be compared with an appropriate indicator-free or low-expression control.

Synthetic dyes introduce different uncertainties. Rhod-2 can show incomplete mitochondrial targeting, cytosolic retention, leakage, and variable de-esterification; accumulation of the cationic dye also depends partly on membrane potential (Deak et al., 2021). A change in Rhod-2 fluorescence may therefore reflect matrix Ca^2+^, depolarization, dye loading, mitochondrial mass, or several of these factors. Localization should be demonstrated with an independent mitochondrial marker, and background, photobleaching, pH sensitivity, and mitochondrial mass should be assessed.

### 8.2 Calibration and quantitative reporting

Raw fluorescence and normalized change are not equivalent to Ca^2+^ concentration. Where feasible, minimum and maximum signals and the effective dissociation constant should be established under conditions approximating the target compartment. Published CEPIA protocols used permeabilized cells, defined Ca^2+^ buffers, ionomycin, and organelle-appropriate pH to estimate dynamic range and affinity (Suzuki et al., 2014; Kanemaru et al., 2020). Such calibration must be revalidated in oocytes because permeabilization or ionophore treatment can alter pH, membrane potential, and organelle integrity.

For single-wavelength probes, absolute concentration may remain uncertain; results should then be reported explicitly as background-corrected fluorescence or change from a defined baseline, not in concentration units. Ratiometric probes reduce sensitivity to loading and optical path length but still require calibration, localization validation, and confirmation that neither channel is saturated.

Minimum reporting should include five core measures: resting signal, peak or change from baseline, area under the curve, recovery half-time, and spatial heterogeneity. Rise time and event frequency should be added where applicable. Reports should specify the probe construct or dye, concentration or expression strategy, loading and de-esterification conditions, excitation power, sampling interval, background and bleaching correction, segmentation method, normalization denominator, and the number of donors and experimental batches. Arbitrary fluorescence units should not be compared across probes, imaging systems, or batches without a shared calibration standard.

### 8.3 Bioenergetic, redox, and structural measurements

Ca^2+^ imaging should be integrated with mitochondrial membrane potential, respiration, ATP, redox state, ROS, morphology, and reproductive outcomes. Membrane potential supplies the electrochemical driving force for MCU-mediated entry; a small matrix response in a depolarized oocyte therefore does not by itself indicate reduced MCU abundance or conductance.

JC-1, TMRE, and TMRM signals are influenced by loading, mitochondrial mass, dye concentration, quenching, and imaging conditions and should not be treated as respiration measurements. Respirometry can distinguish basal, ATP-linked, maximal, and reserve capacity, although oocyte measurements may require pooling. The number of oocytes and females, pooling and allocation strategy, normalization method, and number of independent runs should be reported.

ATP reporters can resolve acute energetic changes during maturation or fertilization. NAD(P)H and flavoprotein autofluorescence report cellular redox responses but are not specific assays of Ca^2+^-dependent dehydrogenase activity. Interpretation should account for substrate availability, respiratory-chain activity, compartmental contributions, and mitochondrial mass.

ROS indicators likewise require chemical and spatial qualification. DCF-related fluorescence depends on probe localization, light exposure, antioxidant activity, and the oxidant species present; it should not be called mitochondrial ROS without mitochondrial targeting and appropriate specificity controls. Mitochondrial targeting alone does not make a probe specific for a single reactive species.

Mitochondrial morphology and distribution should be quantified with prespecified measures such as area, aspect ratio, branching, circularity, spatial density, and distance from the germinal vesicle or spindle. mtDNA copy number and mitochondrial mass are not substitutes for respiratory competence; increases may reflect biogenesis, swelling, impaired mitophagy, or compensation for poor function.

### 8.4 Biochemical uptake assays and electrophysiology

Mitochondrial Ca^2+^ transport can be examined in isolated mitochondria, permeabilized or intact cultured cells, and mitoplasts with fluorescent uptake assays or electrophysiology (Deak et al., 2021). Radionuclide uptake and heterologous electrophysiological reconstruction provide additional quantitative approaches (Rodriguez et al., 2021). These reductionist methods resolve kinetics that endpoint imaging in intact oocytes cannot provide.

Radionuclide uptake in cultured cell lines measures uniporter transport quantitatively, whereas expression in *Xenopus* oocytes permits electrophysiological analysis of mutations, ion selectivity, inhibitor sensitivity, and transport kinetics independently of downstream reproductive phenotypes (Rodriguez et al., 2021).

Plasma-membrane targeting of human MCU and EMRE in *Xenopus* oocytes produced inwardly rectifying Ca^2+^ currents that were blocked by Ru360. Mutations disrupting MCU-EMRE interaction or the pore Ca^2+^-binding site abolished the recorded currents (Tsai and Tsai, 2018).

This system establishes channel properties but does not reproduce the native inner-membrane potential, lipid and matrix environment, contact-site architecture, or accessory-subunit stoichiometry of an oocyte. Conclusions from heterologous systems should therefore be tested with native matrix imaging, bioenergetic measurements, and reproductive outcomes.

### 8.5 Establishing causality and statistical independence

Ruthenium red and Ru360 are widely used to restrict mitochondrial Ca^2+^ uptake, but neither establishes MCU-specific causality alone. Ruthenium red affects multiple Ca^2+^-permeable channels and has variable cellular permeability; Ru360 is more selective for the uniporter in reductionist recordings but may enter intact cells inefficiently (Deak et al., 2021; Tsai and Tsai, 2018). Dose, exposure duration, cell state, and confirmation of the matrix Ca^2+^ response are therefore essential when interpreting reproductive experiments such as those using mouse or porcine oocytes (Wang et al., 2021b; Zhang et al., 2022; Jegal et al., 2020).

CGP37157 and erastin should be treated as non-selective pharmacological perturbations rather than as target-specific probes in reproductive experiments (Wang et al., 2021b). CGP37157 can affect multiple Ca^2+^-handling processes, whereas erastin has broader effects on cellular redox metabolism and ferroptosis. IP_3_R inhibitors, chelators, ionophores, and plasma-membrane channel blockers likewise alter several parts of the cellular Ca^2+^ network. Results obtained with one compound should be confirmed by a mechanistically independent intervention.

A strong causal design combines acute pharmacology with cell-type-specific genetic manipulation, transporter rescue, and an orthogonal Ca^2+^ assay. Inducible models are preferable to constitutive deletion when developmental compensation or systemic effects are plausible, and rescue constructs should distinguish channel conductance from scaffolding or broader changes in mitochondrial state.

The evidentiary sequence should be prespecified. Transporter manipulation should first change a defined mitochondrial Ca^2+^ parameter in the predicted direction; that change should precede the proposed energetic, redox, cytoskeletal, or developmental effect; and direction-appropriate rescue should normalize both Ca^2+^ handling and reproductive function.

Direction-appropriate rescue may mean reducing entry in an overload model, restoring entry in an uptake-deficiency model, or enhancing efflux when recovery is impaired. Improvement in ATP or ROS without demonstrating correction of the prespecified Ca^2+^ abnormality supports a protective effect but leaves the proposed Ca^2+^ mechanism unresolved.

As a reporting standard, this review recommends defining statistical independence according to how treatment was randomized and applied. Multiple oocytes or embryos from one female or human donor are clustered observations, not automatically independent biological replicates. When treatment is assigned within a donor, analyses should model donor as a block or random effect and account for culture drop or dish, batch, and repeated imaging; when treatment is assigned to the female, the female is the experimental unit. Reports should distinguish numbers of donors, independent experimental runs, pools, and individual oocytes and should include blinding, prespecified exclusions, and all measured developmental outcomes.

Table 3 summarizes the resulting minimum framework for Ca^2+^ dynamics, multi-organelle imaging, mitochondrial function, causal perturbation, reproductive outcomes, long-term safety, and donor-aware statistical design.

**TABLE 3:** Recommended experimental framework.

Domain	Minimum recommended measurements
Ca^2+^ dynamics	Resting matrix Ca^2+^, peak, area under the curve, recovery half-time, and spatial heterogeneity
Multi-organelle signaling	Simultaneous cytosolic, ER, matrix, and contact-site Ca^2+^
Mitochondrial function	Membrane potential, oxygen consumption, ATP, NAD(P)H, ROS, morphology, and distribution
Causal perturbation	Acute pharmacology, cell-specific genetics, transporter rescue, and orthogonal validation
Reproductive outcomes	Meiotic progression, spindle integrity, aneuploidy, fertilization, cleavage, and blastocyst formation
Long-term safety	Implantation, placental function, fetal growth, postnatal health, fertility, and, where feasible in preclinical models, multigenerational outcomes
Statistical design	Donor-aware biological replication, experimental-unit definition, nested analysis, blinding, and prespecified exclusions

## 9 Pharmacological interventions and translational relevance

The non-linear relationship between mitochondrial Ca^2+^ and reproductive function creates a state-dependent therapeutic problem. An intervention that benefits an oocyte with matrix Ca^2+^ overload may impair an oocyte in which physiological uptake is required for ATP production. Translational assessment must therefore define the baseline Ca^2+^ phenotype, developmental stage, exposure duration, and molecular target before treatment.

### 9.1 Inhibition of mitochondrial Ca^2+^ uptake

Inhibition of mitochondrial Ca^2+^ uptake may be beneficial when excessive or prolonged matrix loading has been demonstrated. In vitrified germinal-vesicle-stage mouse oocytes undergoing subsequent *in-vitro* maturation, Ru360 lowered the mitochondrial Ca^2+^ signal and improved mitochondrial function, spindle organization, chromosome alignment, and meiotic progression (Sun et al., 2023). These findings support intervention in a defined cryopreservation-associated model, not routine inhibition across oocytes or developmental stages.

In porcine zygotes, 20 μM ruthenium red reduced Rhod-2 fluorescence and MICU1 protein abundance and increased blastocyst formation, whereas 10 and 40 μM did not improve blastocyst yield (Jegal et al., 2020). This restricted dose response does not establish that the physiological post-fertilization mitochondrial Ca^2+^ increase is pathological or that MCU inhibition caused the developmental benefit.

Neither uncalibrated Rhod-2 fluorescence nor MICU1 abundance is a direct quantitative measure of matrix Ca^2+^ concentration or uptake rate. Ruthenium red also has variable cellular permeability and off-target actions (Deak et al., 2021; Tsai and Tsai, 2018). The porcine findings therefore do not provide evidence of MCU-specific rescue.

The same intervention may be harmful when physiological uptake is required for metabolic activation. MCU inhibition or knockdown reduced ATP production and impaired meiotic progression in otherwise unstressed mouse oocytes (Zhang et al., 2021). Treatment effects consequently depend on baseline matrix Ca^2+^, mitochondrial membrane potential, developmental stage, dose, and exposure duration.

A short exposure during cryoprotectant loading or warming may limit an acute Ca^2+^ increase, whereas persistent inhibition during recovery, maturation, or fertilization may deprive the oocyte of necessary metabolic stimulation. Studies should therefore compare narrowly defined exposure windows and require quantitative evidence that treatment corrects the baseline abnormality. Empirical treatment of all oocytes with an MCU inhibitor is not supported by current evidence.

### 9.2 Pleiotropic protective compounds

Several natural or endogenous compounds improve ovarian or oocyte phenotypes while modifying Ca^2+^-related endpoints, but their evidence ranges from pathway-level experiments to cytosolic fluorescence or gene-expression associations. These levels should not be interpreted as equivalent evidence of mitochondrial transporter regulation.

In DHEA-induced PCOS mouse and granulosa-cell models, puerarin reduced cytosolic Ca^2+^ accumulation and RyR- and IP_3_R-associated Ca^2+^ release. Promoter-reporter and pathway-inhibition experiments linked calcineurin-NFATc signaling to Mcu expression, while mitochondrial Ca^2+^, ATP, membrane-potential, and permeability-transition-related endpoints also improved (Wang et al., 2024). This provides pathway-level evidence involving MCU but does not show that puerarin directly binds or selectively modulates the uniporter.

At the organelle-fluorescence level, salidroside improved cytosolic and Rhod-2-associated mitochondrial Ca^2+^ signals, mitochondrial distribution, membrane potential, ATP, redox balance, spindle organization, and developmental competence in postovulatory-aged mouse oocytes (Liu et al., 2024). Melatonin likewise reduced oxybenzone-associated cytosolic and mitochondrial Ca^2+^ signals while improving mitochondrial dynamics, membrane potential, electron-transport-related gene expression, redox balance, and spindle organization (Shi et al., 2025). Both compounds are pleiotropic, and neither study established calibrated uptake or efflux kinetics or MCU-specific target engagement.

In H_2_O_2_-exposed porcine granulosa cells, 1-deoxynojirimycin reduced Rhod-2 fluorescence attributed to mitochondrial Ca^2+^, mitochondrial ROS, ER stress, and apoptosis while modifying PERK-ATF4/MFN2-associated MAM signaling (Xing et al., 2025). ATF4 knockdown attenuated several related abnormalities, supporting involvement of ER-stress-MAM signaling; however, the work used cultured porcine granulosa cells and did not provide *in-vivo* reproductive validation or calibrated Ca^2+^ flux measurements.

At a less direct level, apigenin improved ovarian and follicular phenotypes in aged laying hens and an avian follicular-cell senescence model while modulating the expression of several Ca^2+^-handling genes and attenuating ER stress (Gao et al., 2026). That study measured cytosolic Ca^2+^ and gene expression but did not directly quantify mitochondrial Ca^2+^ or demonstrate mitochondrial transporter regulation.

Collectively, these compounds are not established selective modulators of MCU, MICU1, MICU2, or NCLX. Restoration of Rhod-2 or another Ca^2+^-sensitive fluorescence endpoint does not by itself demonstrate direct transporter regulation, calibrated matrix Ca^2+^ concentration, or altered Ca^2+^ flux. Natural origin likewise does not establish specificity, pharmacokinetic suitability, therapeutic efficacy, or reproductive safety.

### 9.3 Biomarker development

Mitochondrial Ca^2+^ remains a candidate research biomarker rather than a validated basis for selecting oocytes, embryos, or patients. Resting matrix Ca^2+^, peak amplitude, area under the curve (integrated exposure), recovery half-time, and spatial heterogeneity are distinct candidate variables; a single high or low fluorescence value cannot distinguish excessive uptake, impaired efflux, altered buffering, or loss of signal caused by depolarization. Because direct measurements in viable human oocytes are invasive and technically demanding, cumulus or granulosa cells and follicular fluid may be evaluated as surrogates only after concordance with the oocyte compartment has been demonstrated (Yildirim and Seli, 2024a).

Development of a clinically useful biomarker requires a prespecified assay and threshold, calibration and batch controls, donor-level separation of training and validation datasets, and external validation across centers and platforms. Predictive value should be tested beyond maternal age, ovarian diagnosis, oocyte morphology, embryo morphokinetics, and, where available, ploidy. Analyses must account for clustering of multiple oocytes or embryos from one patient and should report calibration, discrimination, reproducibility, and decision-relevant improvement rather than an isolated association with blastocyst formation.

### 9.4 Direct clinical manipulation

Direct transporter manipulation should be considered only after distinguishing deficient uptake, excessive or prolonged loading, impaired efflux, and secondary loss of mitochondrial Ca^2+^ responsiveness. Enhancing uptake may support ATP production in an oocyte with demonstrated uptake deficiency but worsen injury when matrix Ca^2+^ is already excessive; inhibition may protect during a defined cryopreservation-associated increase while impairing physiological meiotic or fertilization-associated metabolic activation.

Age, obesity, PCOS, and oxidative stress are heterogeneous clinical or experimental contexts, not surrogate diagnoses of mitochondrial Ca^2+^ overload; reported Ca^2+^-associated phenotypes differ by model, compartment, and assay (Zhao et al., 2017; Wang et al., 2024; Gao et al., 2026; Xing et al., 2025). Stratification should therefore identify the measured transport or recovery defect rather than infer it from the diagnostic label.

Transporter-directed treatment would require patient or oocyte stratification, direct evidence of target engagement, and a short, developmentally defined exposure window. Dose-response and washout studies should demonstrate reversibility and absence of persistent activity in later embryonic stages.

The literature reviewed here does not establish a clinically validated MCU-, MICU-, or NCLX-directed treatment for assisted reproduction. Changes in ATP, ROS, morphology, meiotic progression, or blastocyst formation are insufficient unless the intended transporter and Ca^2+^ parameter change in the predicted direction. Safety assessment should extend beyond early developmental endpoints to chromosome segregation, epigenetic reprogramming, implantation, placental and fetal development, postnatal health, fertility, and, where feasible, multigenerational outcomes.

Mitochondrial replacement and related cytoplasmic interventions demonstrate that ooplasmic quality can be therapeutically relevant, but they do not provide direct evidence for mitochondrial Ca^2+^ pharmacology and raise distinct genetic, ethical, and regulatory issues (Yildirim and Seli, 2024b).

### 9.5 Oxidative stress, mitochondrial permeability transition, and the ROS–Ca^2+^ interlock

Matrix Ca^2+^ loading and oxidative stress can form a bidirectional amplification loop rather than a fixed linear sequence. Excessive matrix Ca^2+^ can disrupt respiratory function, increase ROS, and sensitize the mitochondrial permeability transition pore (mPTP), whereas ROS and ER stress can alter contact-site Ca^2+^ release, transporter abundance, and membrane potential, thereby changing subsequent uptake and efflux (Zhang et al., 2020; Wang et al., 2024; Xing et al., 2025; Bonora et al., 2022). These associations do not establish that Ca^2+^ overload is invariably the initiating lesion.

mPTP opening is a downstream convergence point for Ca^2+^ loading, redox stress, and loss of membrane potential, but elevated ROS, depolarization, and increased Ca^2+^-sensitive fluorescence may arise together (Bonora et al., 2022; Halestrap and Richardson, 2015). Intervention studies should therefore combine calibrated or ratiometric matrix Ca^2+^ measurements and uptake-recovery kinetics with ROS, membrane-potential, pore-opening, bioenergetic, and developmental readouts. Rescue by an antioxidant or an mPTP-modifying intervention alone does not prove that altered mitochondrial Ca^2+^ transport was the primary therapeutic target.

## 10 Evidence gaps and future directions

Three evidence gaps dominate the field.

First, direct, compartment-resolved measurements of mitochondrial Ca^2+^ dynamics in viable human oocytes remain scarce. Available human experiments frequently rely on donated oocytes that are not intended for clinical transfer (Yildirim and Seli, 2024a; Yildirim and Seli, 2024b). Findings from mouse and porcine oocytes cannot be assumed to translate directly because oocyte physiology, fertilization-associated Ca^2+^ signaling, and developmental timing differ among species (Yildirim and Seli, 2024a; Nagaraju et al., 2026).

Second, the stage- and cell-type-resolved expression, localization, stoichiometry, and assembly of MCU, MCUb, EMRE, MICU1, MICU2, NCLX, and TMEM65 remain incompletely mapped during follicular growth, meiotic maturation, fertilization, and preimplantation development (Fan et al., 2025; Garbincius et al., 2025; Meng et al., 2023; Lan et al., 2022). Evidence from isolated stages, individual cell types, or heterologous systems does not define the native transporter complexes throughout the reproductive trajectory.

Third, free matrix Ca^2+^ concentration, total mitochondrial Ca^2+^ content, influx and efflux kinetics, buffering, spatial distribution, and integrated exposure are non-equivalent and are often not clearly distinguished (Deak et al., 2021; Suzuki et al., 2014; Kanemaru et al., 2020; Rodriguez et al., 2021). Minimum dynamic reporting should use the same five core measures applied in Section 8.2 and Table 3: resting matrix Ca^2+^, peak amplitude, area under the curve, recovery half-time, and spatial heterogeneity. Rise time and event frequency should be added where applicable.

A staged roadmap follows from these gaps. (i) Reference Ca^2+^ maps should span follicular development, maturation, fertilization, and cleavage, with stage-, cell-type-, and species-resolved measurements. (ii) Acute or inducible, cell-type-specific genetic models should distinguish mitochondrial Ca^2+^ functions in oocytes, cumulus cells, mural granulosa cells, and theca cells. (iii) Multi-compartment imaging of the cytosol, ER, mitochondrial matrix, and contact sites should be integrated with bioenergetic and redox readouts (Suzuki et al., 2014; Kanemaru et al., 2020; Olszakier et al., 2025). (iv) Clinical diagnoses and experimental stressors should be prespecified and analyzed as distinct models rather than pooled; the contexts summarized in Figure 5 should not be treated as a single mitochondrial Ca^2+^ phenotype.

Human studies should define the patient or donor as the clustering unit and distinguish the numbers of oocytes, women, and independent experimental runs. Essential documentation includes maternal age, ovarian diagnosis, stimulation protocol, oocyte maturation stage, fertilization method where applicable, culture medium, retrieval-to-measurement interval, and the reason for each exclusion. Biomarker analyses should follow the assay, validation, clustering, and incremental-value requirements described in Section 9.3, with ploidy considered only when it is available and relevant to the intended use.

Finally, preclinical germ-cell manipulation studies should extend beyond GVBD, first-polar-body extrusion, cleavage, and blastocyst formation to evaluate chromosome segregation and embryo ploidy, implantation, placental and fetal development, postnatal health and fertility, and, where feasible, multigenerational outcomes. Human studies should instead use ethically appropriate longitudinal follow-up of offspring and should not imply experimental multigenerational testing.

## 11 Conclusion

Mitochondrial Ca^2+^ homeostasis provides a dynamic interface between Ca^2+^ signaling and mitochondrial metabolism in ovarian cells and oocytes. Cytosolic signals generated by plasma-membrane Ca^2+^ entry and ER/IP_3_R1 release can be transferred at ER–mitochondria contacts, pass the outer membrane through VDAC1, and enter the matrix through the MCU complex (Baughman et al., 2011; Tsai and Tsai, 2018; Kamer et al., 2019; Zhang et al., 2024; Savy et al., 2022; Wang et al., 2021a; Kang et al., 2023). Within a physiological range, matrix Ca^2+^ supports TCA-cycle activity, oxidative phosphorylation, and ATP supply, whereas excessive or prolonged loading increases susceptibility to oxidative injury and mitochondrial permeability transition (Szabadkai et al., 2001; Kamer and Mootha, 2015; Zhang et al., 2020; Bonora et al., 2022). NCLX mediates mitochondrial Ca^2+^ efflux and may shape recovery between successive transients, but its proposed role in decoding fertilization-associated oscillations has not been tested directly; TMEM65 supports NCLX in non-reproductive systems, and its ovarian role remains unknown (Fan et al., 2025; Garbincius et al., 2025; Meng et al., 2023).

The relationship between mitochondrial Ca^2+^ and reproductive outcome is therefore non-linear and context-dependent. Loss or inhibition of MCU-dependent uptake reduces ATP production and impairs meiotic progression in otherwise unstressed mouse oocytes (Zhang et al., 2021), whereas overload models link increased matrix Ca^2+^ to oxidative injury, spindle and chromosome defects, and reduced developmental competence (Zhang et al., 2020; Sun et al., 2023). Maternal NLRP14-dependent regulation of NCLX provides one of the strongest reproductive genetic links between mitochondrial Ca^2+^ homeostasis and the oocyte-to-embryo transition (Meng et al., 2023), while the stage-specific contribution of uptake, efflux, buffering, and membrane potential remains to be resolved.

Current evidence is strongest in animal oocytes, embryos, and experimentally manipulated follicular cells; direct, calibrated evidence from viable human oocytes remains insufficient (Yildirim and Seli, 2024a; Yildirim and Seli, 2024b). The pore-forming architecture of the mitochondrial permeability transition pore also remains unresolved: F1Fo ATP synthase is strongly implicated, but adenine nucleotide translocase, the phosphate carrier, and associated inner-membrane complexes remain part of competing or overlapping models (Bonora et al., 2022; Halestrap and Richardson, 2015). Resolving this architecture will influence the design and interpretation of Ca^2+^-overload interventions but will not by itself establish reproductive efficacy or safety. Until compartment-resolved human data, transporter-specific causality, and long-term developmental validation are available, mitochondrial Ca^2+^ should be treated as a mechanistic research axis with translational potential rather than a validated clinical target.

## Glossary

AKT: protein kinase B
AMPK: AMP-activated protein kinase
AOA: artificial oocyte activation
ATF4: activating transcription factor 4
ATP: adenosine triphosphate
BAPTA: 1,2-bis(o-aminophenoxy)ethane-N,N,N′,N′-tetraacetic acid
Bax: Bcl-2-associated X protein
Bcl-2: B-cell lymphoma 2
CaMKK2: calcium/calmodulin-dependent protein kinase kinase 2
cAMP: cyclic adenosine monophosphate
**Ca** ^**2+**^: calcium ion
CCCP: carbonyl cyanide m-chlorophenyl hydrazone
CEPIA: calcium-measuring organelle-entrapped protein indicators
COC: cumulus-oocyte complex
CsA: cyclosporin A
CypD: cyclophilin D
Cyt c: cytochrome c
DCF: 2′,7′-dichlorofluorescein
DHEA: dehydroepiandrosterone
DIME: Asp-Ile-Met-Glu selectivity motif
eIF4E: eukaryotic translation initiation factor 4E
EMRE: essential MCU regulator
ER: endoplasmic reticulum
ERK: extracellular signal-regulated kinase
F1Fo: ATP synthase F1 and Fo sectors
FAD: flavin adenine dinucleotide
FOXJ2: forkhead box J2
GRP75: glucose-regulated protein 75
GV: germinal vesicle
GVBD: germinal-vesicle breakdown
HB-EGF: heparin-binding EGF-like growth factor
**H**_**2**_ **O**_**2**_: hydrogen peroxide
ICSI: intracytoplasmic sperm injection
IMM: inner mitochondrial membrane
IMS: intermembrane space
**IP**_**3**_: inositol 1,4,5-trisphosphate
**IP**_**3**_ **R1**: inositol 1,4,5-trisphosphate receptor type 1
IRE1α: inositol-requiring enzyme 1 alpha
IVF: *in vitro* fertilization
JC-1: 5,5′,6,6′-tetrachloro-1,1′,3,3′-tetraethylbenzimidazolocarbocyanine iodide
KGN: human granulosa-like tumor cell line
KO: knockout
LH: luteinizing hormone
MAM: mitochondria-associated membrane
MAPK: mitogen-activated protein kinase
MCU: mitochondrial calcium uniporter
MCUb: mitochondrial calcium uniporter b
MFN2: mitofusin 2
MII: metaphase II
mPTP: mitochondrial permeability transition pore
mtDNA: mitochondrial DNA
mTOR: mechanistic target of rapamycin
NAD(P)H: reduced nicotinamide adenine dinucleotide (phosphate)
NCLX: Na^+^/Ca^2+^–Li^+^ exchanger
NFATc: nuclear factor of activated T cells, cytoplasmic
NLRP14: NLR family pyrin domain containing 14
NMBR: neuromedin B receptor
OMM: outer mitochondrial membrane
OSCP: oligomycin sensitivity-conferring protein
PACS-2: phosphofurin acidic cluster sorting protein 2
PB1: first polar body
PCOS: polycystic ovary syndrome
PDH: pyruvate dehydrogenase
PERK: protein kinase R-like endoplasmic reticulum kinase
PLCζ: phospholipase C zeta
PMCA: plasma membrane Ca^2+^ ATPase
PMCA1: plasma membrane Ca^2+^ ATPase 1
POI: premature ovarian insufficiency
PTP: permeability transition pore
ROS: reactive oxygen species
RyR: ryanodine receptor
SD: standard deviation
SEM: standard error of the mean
SERCA: sarco/endoplasmic reticulum Ca^2+^ ATPase
siRNA: small interfering RNA
SLC8B1: solute carrier family 8 member B1
StAR: steroidogenic acute regulatory protein
TCA: tricarboxylic acid cycle
TMEM65: transmembrane protein 65
TMRE: tetramethylrhodamine ethyl ester
TMRM: tetramethylrhodamine methyl ester
TRPM7: transient receptor potential melastatin 7
TSPO: translocator protein
VDAC1: voltage-dependent anion channel 1
WGA: wheat germ agglutinin
ZGA: zygotic genome activation
ΔΨm: mitochondrial membrane potential.
